# Synthesis, Hydrolytic Degradation Behavior, and Surface Properties of Poly(alkyl glycolide)-Polyglycolide Copolymers

**DOI:** 10.1021/acsomega.4c10768

**Published:** 2025-02-20

**Authors:** Mehtap Cantürk Bamyacı, Duygu Çetin, Candan Cengiz, Sema Nur Belen, Olcay Mert, Ugur Cengiz, Serap Mert

**Affiliations:** † Department of Polymer Science and Technology, Kocaeli University, 41001 Kocaeli, Turkey; ‡ AFC Green Technologies R&D, Çanakkale Technopark, Sarıcaeli, 17100 Çanakkale, Turkey; ^§^Department of Energy Resources and Management, Faculty of Engineering, ^¶^Surface Science Research Laboratory, Department of Chemical Engineering, Faculty of EngineeringÇanakkale Onsekiz Mart University, 17100 Çanakkale, Turkey; ∥ Department of Chemistry, Kocaeli University, 41001 Kocaeli, Turkey; ⊥ Center for Stem Cell and Gene Therapies Res. and Pract., Kocaeli University, 41001 Kocaeli, Turkey; # Department of Chemistry and Chemical Processing Tech., Kocaeli University, 41140 Kocaeli, Turkey

## Abstract

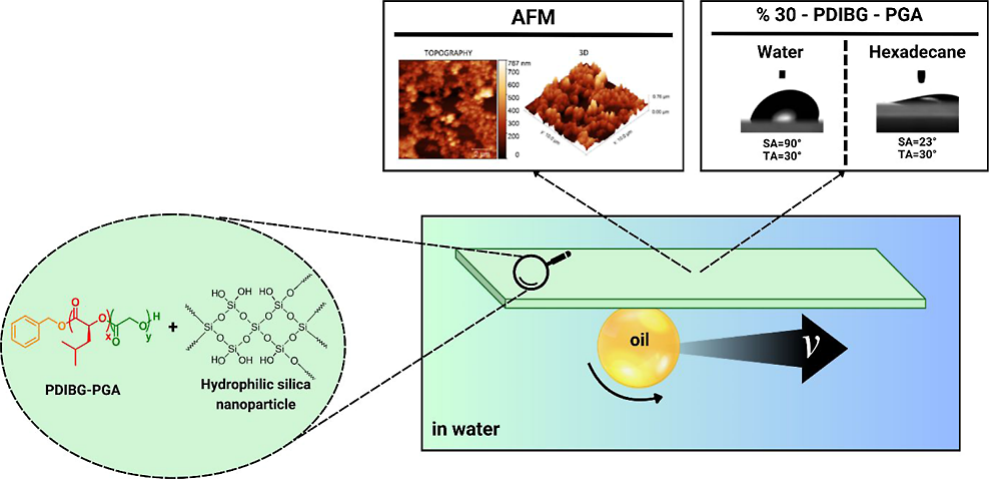

Given the environmental impact of polymers on our daily lives, the development of biodegradable polymers is becoming increasingly critical. Poly(diisobutyl glycolide)–polyglycolide (PDIBG–PGA) and poly(diisopropyl glycolide)-polyglycolide (PDIPG–PGA) copolymers, which are structurally similar to polylactic-*co*-glycolic acid (PLGA) polyesters frequently used in the field of biomaterials, were synthesized via ring-opening polymerization (ROP) of glycolide with l-diisobutyl glycolide (l-DIBG) or l-diisopropyl glycolide (l-DIPG), respectively, in various molecular weights (*M*_w_^GPC^: 15.5–40.0 kDa) and in high yields (up to 85.0%). The wettability characteristics of biodegradable polymers are important not only in air but also for their behavior in underwater environments. PDIBG–PGA silica composites, due to their amphiphilic nature, exhibited water contact angles between 72° and 85° in air, unaffected by the increasing addition of hydrophilic silica nanoparticles. However, underwater–oil contact angles increased from 75° to 165° as a result of the higher silica nanoparticle content and enhanced surface roughness. When the silica content reached 30%, the surface demonstrated self-cleaning and oil-repellent properties underwater, attributed to the Cassie state, which trapped air within the surface’s hierarchical roughness. Furthermore, the surface free energy (SFE) values of PDIBG-PGA and PDIPG-PGA copolymer films were evaluated using the Owens-Wendt method, which revealed an increasing underwater hexadecane contact angle as the polar component interactions increased. Differential scanning calorimetry analysis revealed that all synthesized copolymers were amorphous, and the glass transition temperatures (*T*_g_) increased with the increase in the molecular weight of the copolymers (for instance, *M*_n_^GPC^: 9560 g/mol → *T*_g_: 25.1 °C vs *M*_n_^GPC^: 20,850 g/mol → *T*_g_: 32.3 °C for PDIBG–PGA; *M*_n_^GPC^: 10,670 g/mol → *T*_g_: 37.7 °C vs *M*_n_^GPC^: 23,360 g/mol → *T*_g_: 42.3 °C for PDIPG–PGA). The molecular weight decreases of 88.3% and 76.5% and mass losses of 36.7% and 12.3% were observed for PDIBG–PGA and PDIPG–PGA copolymers after 8 weeks of hydrolytic degradation, respectively. The faster degradation of PDIBG–PGA (*T*_g_: 25.1 °C) than PDIPG–PGA (*T*_g_: 37.7 °C) may be attributed to the *T*_g_ below the hydrolytic degradation temperature (37 °C) because of an increase in the mobility of PDIBG–PGA polymer chains, allowing water molecules to transfer more easily through the matrix.

## Introduction

Due to the limited supply of fossil fuels and environmental concerns, the attention of researchers is increasingly shifting to biodegradable polymers that can be decomposed into water and CO_2_ through microbial action in typical ecological cases.^1^ These polymers offer many advantages, such as not causing systemic toxicity when used in controlled drug delivery system applications and in the development of environmentally friendly products.^2,3^ PLGA, polylactide (PLA), and PGA are among the most preferred polymers in biomedical applications due to their superior properties such as biodegradability, biocompatibility, and low toxicity for the treatment of various diseases^4^ such as cancer,^5^ cardiovascular,^6^ inflammatory,^7^ and cerebral^8^ diseases, as well as bone repair^9,10^ and surgical sutures.^11,12^ It has been reported that only 50% of semicrystalline PLLA homopolymers degrade within 1–2 years, while amorphous poly(glycolic acid-*co*-l-lactic acid) copolymers, synthesized by introducing crystalline PGA blocks into the homopolymer, completely degrade within 50–100 days.^13^ Poly(substituted glycolides), which are structurally similar to PLA^1^ and PLGA polyesters,^14^ have attracted a lot of attention due to properties such as ring-opening reactivity^15−17^ of their constituent monomers, eletrospun fiber films,^17^ degradability,^15−24^ and biocompatibility.^15^

PEG-based amine-functionalized polylactide-poly(amino ethyl methyl glycolide) (P(LLA-NEtMG)) diblock and triblock copolymers with various molecular weights (*M*_n_^GPC^: between 3780 and 5750 g/mol) for local chemotherapy applications were obtained with high conversions (up to 96%) and low PDI values (between 1.06 and 1.27) in the presence of tin octoate (Sn(Oct)_2_) catalyst in only 1 h of reaction time. Hydrolytic degradation studies carried out at pH:7.4 and 37 °C for 1 month showed that amine-functionalized PEG based diblock (47% degradation) and triblock (28% degradation) copolymers degraded faster than highly crystalline and hydrophobic classical PEG-based PLLA diblock (0.6% degradation) and triblock (13.8% degradation) copolymers. The faster degradation of functional diblock and triblock copolymers was attributed to the breakdown of crystallinity in the presence of NEtMG and the presence of hydrophilic amine groups in the copolymers.^22^

Poly(monohexyl-substituted glycolide) (PmHSG) homopolymers were synthesized by the ring-opening polymerization (ROP) in the presence of a benzyl alcohol initiator and Sn(Oct)_2_ catalyst, and the effect of the introduction of hexyl side groups into the polymer skeleton on the hydrolytic degradation rate was evaluated by comparing with the degradation rate of conventional PLA polymers. It was predicted that the degradation rate of PmHSG would be slower compared to PLA due to the higher steric hindrance and hydrophobicity of the hexyl side groups. However, PmHSG (*T*_g_: ∼−15 °C) was found to degrade slightly faster than PLA (*T*_g_: ∼ 40 °C) in the hydrolytic degradation study carried out at pH 7.4 and 37 °C for 7 weeks, and this was attributed to the flexible rubbery state of PmHSG at the degradation temperature due to its low *T*_g_.^20^

Poly(l-lactic acid-*co*-(*S*)-2-hydroxy-4-methylpentanoic acid) (P(LLA-*co*-LLOH)) and poly(l-lactic acid-*co*-(*S*)-2-hydroxy-3-methylbutanoic acid) (P(LLA-*co*-LVOH)) copolymers were synthesized by polycondensation of LLA with LLOH or LVOH with the elimination of water at different feeding rates (LLA/LLOH (mol^NMR^ %): 99/1, 94/6, 89/11; LLA/LVOH (mol^NMR^ %): 98/2, 95/5, 90/10), and then, the enzymatic degradation behavior of the prepared copolymer films was investigated. It was found that an increase in the content of side chain substituted lactic acid (from 1 to 6 mol for LLOH) in these copolymer films speeded up the enzymatic degradation by proteinase K.^21^

A series of PDIPG-mPEG diblock and PDIPG–PEG-PDIPG triblock copolymers were synthesized in various molecular weights (*M*_n_^GPC^: between 2930 and 16,000 g/mol) by the ROP in the presence of Sn(Oct)_2_ catalyst with conversions as high as 99.9% and narrow polydispersity indexes as low as 1.04 for use in local drug delivery systems. PDIPG–PEG-PDIPG triblock copolymer was reported to degrade to oligomeric species faster than the PDIPG-mPEG diblock copolymer (26% vs 15%) at 37 °C at the end of 2 weeks.^23^ PDIBG-mPEG diblock and PDIBG–PEG-PDIBG triblock copolymers were also synthesized in a similar methodology above at high conversions up to 99.2%, narrow PDI values as low as 1.11, and desired molecular weights (*M*_n_^GPC^: between 3920 and 15,590 g/mol) close to the targeted molecular weights. Then, their paclitaxel anticancer drug-loaded nanoparticles were prepared, and their hydrolytic degradation behaviors at 37 and 55 °C were evaluated by gel permeation chromatography (GPC) analyses. It was determined that diblock copolymer-based nanoparticles (*M*_n_^GPC^ loss: 46.7% at 37 °C and 93.1% at 55 °C) degraded faster than triblock copolymer-based nanoparticles (*M*_n_^GPC^ loss: 34.0% at 37 °C and 82.9% at 55 °C) at the end of 1 month at both temperature values. The differences in their degradation rates were attributed to the position of the PEG segment (lateral or internal) in the copolymer. In addition, the higher molecular weight loss of both diblock (*T*_g_: 16.4 °C) and triblock (*T*_g_: 10.9 °C) copolymers at 55 °C compared to 37 °C is due to the easier transfer of water through the matrix with increased chain mobility at 55 °C, a temperature well above *T*_g_ values of polymers.^16^

A series of mPEG-poly(isobutyl methyl glycolide) diblock and poly(isobutyl methyl glycolide)-PEG-poly(isobutyl methyl glycolide) triblock copolymers were synthesized with high conversions (up to 99.0%) and narrow PDI values (as low as 1.08), and then, the formation of thermosensitive gels and hydrolytic degradation behavior were studied. In hydrolytic degradation experiments of diblock and triblock copolymer gels at 37 °C, the diblock copolymer with PEG in the lateral segment (34.6% degradation for PIBMG units) was reported to degrade faster at the end of 4 weeks compared to the triblock copolymer with PEG in the inner segment (23.7% degradation for PIBMG units).^15^

Poly(methyl glycolide), poly(benzyl glycolide), poly(isobutyl glycolide), poly(isopropyl glycolide), and poly(phenyl glycolide) homopolymers were obtained by ROP in the presence of a TBD organocatalyst and benzyl alcohol initiator at different reaction times (0.5, 2, and 18 h) with high conversions (up to 99%) and slightly high PDI values (between 1.41 and 1.92). Then, their fibers were prepared by the electrospinning method, and the hydrolytic degradation behavior of these fibers was determined by following the loss of molecular weight per week for 10 weeks at pH 7.4 and 37.0 °C. When these five homopolymer-based fibers were compared within themselves, it was determined that the degradation rates changed depending on the nature of the substituent in the polymer chain; in other words, poly(methyl glycolide) without steric hindrance (∼70% relative *M*_w_ change at week 10) degraded the fastest, while poly(phenyl glycolide) with the highest steric hindrance (∼5% relative *M*_w_ change at week 10) degraded the slowest. Furthermore, a more linear decrease in molecular weight with time and faster degradation behavior were observed for poly(benzyl glycolide) and poly(isobutyl glycolide) polymers with secondary β carbon atoms compared to poly(isopropyl glycolide) with tertiary β carbon atoms [relative *M*_w_ change at week 10: ∼38% for poly(benzyl glycolide); ∼37% for poly(isobutyl glycolide); and ∼30% for poly(isopropyl glycolide)].^17^

In the current work, for the first time to our knowledge, PDIBG–PGA and PDIPG–PGA copolymers, which are structurally similar to PLGA polyesters, were synthesized in various molecular weights by ROP of glycolide with l-DIBG or l-DIPG monomers in the presence of benzyl alcohol as an initiator and Sn(Oct)_2_ as a catalyst, respectively, and their hydrolytic degradation behaviors and surface characteristics were investigated. When analyzing the wettability behaviors of PDIBG–PGA and PDIPG–PGA copolymer thin films, it is evident that the surfaces exhibit hydrophobic characteristics in air and oleophilic behavior underwater. With the incorporation of hydrophilic silica into the PDIBG–PGA copolymer, which has a higher underwater oleophobicity value than the PDIPG–PGA copolymer, the surfaces demonstrate underwater superoleophobic properties. The PDIBG–PGA composite film with 30 wt % silica also shows moderate dynamic oleophobicity in air, with a sliding angle (SA) of 23°, in addition to its underwater superoleophobicity. This study introduces significant advancements related to PDIBG–PGA-silica composites, particularly in terms of oil-repellent behaviors in both air and underwater environments and their dependence on surface roughness. Moreover, PDIBG homopolymers are known to be highly hydrophobic and therefore very stable polymers against hydrolysis even at 55 °C.^24^ PDIPG has been reported as a semicrystalline homopolymer.^25^ Introducing PGA moieties to PDIPG units led to amorphous PDIPG–PGA. The addition of PGA blocks led to the formation of random order and thus helped the degradation of PDIBG–PGA and PDIPG–PGA backbones. Both PDIBG–PGA and PDIPG–PGA copolymers, which showed a significant decrease in molecular weight in only two months (*M*_n_^GPC^: 9560 → 1120 g/mol for PDIBG–PGA and *M*_n_^GPC^: 10,670 → 2510 g/mol for PDIPG–PGA) at 37 °C, are predicted to be a good alternative to biodegradable PLGA copolymers frequently used in environmental and biomedical applications.

## Experimental Section

### Materials

Sulfuric acid (H_2_SO_4_) (95–97%, Sigma-Aldrich), sodium nitrite (NaNO_2_, Isolab), sodium chloride (NaCl) (>99.8%, Sigma-Aldrich), anhydrous sodium sulfate (Na_2_SO_4_) (Sigma-Aldrich), diethyl ether (99.5%, Sigma-Aldrich), hexane (95%, Sigma-Aldrich), l-Valine (l-2-amino-3-methylbutanoic acid) (98%, Sigma-Aldrich), l-Leucine (l-2-amino-4-methylpentanoic acid) (99%, Sigma-Aldrich), p-toluene sulfonic acid monohydrate (PTSA) (≥98%, Sigma-Aldrich), and toluene (99.5%, Isolab) were used for monomer synthesis. Sn(Oct)_2_ (94.5%, Sigma-Aldrich), benzyl alcohol (>99%, TCI), glycolide (94.5%, Sigma), calcium hydride (CaH_2_) (93%, Acros Organics), dichloromethane (DCM) (≥99%, Isolab), and methanol (≥99.8%, Isolab) were employed in the synthesis and purification of copolymers. DCM was distilled over CaH_2_, and methanol was dried over magnesium and iodine. l-DIBG, l-DIPG, and glycolide were dried by azeotropic distillation at 35 °C in dry toluene, which was previously distilled over sodium metal in the presence of a benzophenone indicator, prior to the copolymerization reactions. The benzyl alcohol initiator was dried by vacuum distillation over CaH_2_. Tetrahydrofuran (THF, 99.9%, HPLC grade) was supplied from Sigma-Aldrich and employed as a mobile phase in the GPC measurements. Ultrapure water, ethylene glycol (EG), formamide, α-bromonaphthalene, methylene iodide, and hexadecane were purchased from Merck. Glass slides (76 × 26 mm, ISOLAB, Türkiye) were employed as substrates.

### Characterization

Structural characterization of the synthesized compounds was carried out by NMR analysis using a Bruker Avance III 400 MHz NMR spectrometer. The functional groups of the compounds were analyzed by an ATR Bruker-Tensor 27 model spectrophotometer in the range 4000–600 cm^–1^. *M*_w_, M_n_ and PDI of the copolymers were determined by GPC. The GPC instrument consisted of a viscotek GPC_max_ autosampler equipped with a pump, a refractive index detector (VE 3580), and a column oven (two columns of 300 × 8 mm Viscotec LT4000L Mixed, Low Org and a guard column of 10 × 4.6 mm Viscotek TGuard). THF was used as the mobile phase, and analyses were carried out at 35 °C at a flow rate of 1 mL min^–1^, an injection volume of 100 μL, and a sample concentration of 12 mg mL^–1^. Nine polystyrene standards in the range of 1.2–400 kDa were used to prepare the calibration curve, and data were obtained by using OmniSEC 5.12 software. Differential scanning calorimetry (DSC) analyses of the copolymers were performed by means of a Mettler Toledo DSC Star System with double heating at a heating and cooling rate of 10 °C min^–1^ under nitrogen atmosphere in the temperature range of −60 to 160 °C. Thermogravimetric analysis (TGA) of the copolymers was carried out using a TGA 1 Star System instrument fed with nitrogen gas at a heating rate of 10 °C min^–1^ from 25 to 600 °C and a volumetric flow rate of 30 mL min^–1^.

### Synthesis of PDIBG–PGA and PDIPG–PGA Copolymers

For the synthesis of PDIBG–PGA **8** copolymer ([*M*_0_]/[*I*_0_]/[*C*_0_]: 40 (20 DIBG + 20 glycolide):1:1), 20.8 mg (0.04851 mmol) Sn(Oct)_2_, 5.3 mg (0.04851 mmol, 5.1 μL) benzyl alcohol, 221.5 mg (0.97037 mmol) DIBG, and 113.8 mg (0.97037 mmol) glycolide were added to a test tube, respectively, and the polymerization was performed in an oil bath at 190 °C for 3 h. PDIBG–PGA **8** copolymer was dissolved in 2 mL of DCM, taken into a centrifuge tube, and 10 mL of methanol was added, and kept overnight at −22 °C. After the cold centrifugation at 12,000 rpm for 2 min, the supernatant was separated via decantation, and PDIBG–PGA **8** copolymer was obtained in 78.1% yield. The synthesis of PDIBG–PGA **9** and **10** copolymers was carried out in 7 h under the above reaction conditions except for increasing the monomer feed ratio from 40 to 80 ([*M*_0_]: 40 DIBG + 40 glycolide) and 120 ([*M*_0_]: 60 DIBG + 60 glycolide), respectively, while keeping the mol of initiator and catalyst constant.^1^H NMR (CDCl_3_, 400 MHz) for PDIBG–PGA **8** δ: 0.86–0.95 (br-s, 6H, 2CH_3_), 0.95–1.03 (d, 6H, 2CH_3_), 1.72–1.91 (m, 6H, 2CH, 2CH_2_), 4.52–4.98 (m, 4H, 2CH_2_), 5.04–5.25 (m, 2H, 2CH), 7.29–7.40 (m, 5H, 5xCH). ^13^C NMR (CDCl_3_, 100 MHz): δ 20.22, 21.76, 23.18, 37.96–39.45, 59.54, 69.97, 165.14–165.49, and 167.73–168.23. CHCl_3_ was fixed to 77.1 ppm. ATR-FTIR (ν_max_/cm^–1^): 2958, 2873 (CH); 1751 (C=O).

For the synthesis of PDIPG–PGA **11** copolymer ([*M*_0_]/[*I*_0_]/[*C*_0_]: 40 (20 DIPG + 20 glycolide):1:1), 20.3 mg (0.04735 mmol) Sn(Oct)_2_, 5.2 mg (0.04735 mmol, 4.973 μL) benzyl alcohol, 189.6 mg (0.9470 mmol) DIPG, and 111.0 mg (0.9470 mmol) glycolide were added to a test tube, respectively, and the polymerization was performed in an oil bath at 180 °C for 3 h. PDIPG–PGA **11** copolymer was dissolved in 2 mL of DCM, taken into a centrifuge tube, and 10 mL of methanol was added and kept overnight at −22 °C. After the cold centrifugation at 12,000 for 2 min, the supernatant was separated via decantation, and PDIPG–PGA **11** copolymer was obtained in 81.0% yield. The synthesis of PDIPG–PGA **12** and **13** copolymers was carried out in 7 h under the same reaction conditions except for increasing the monomer feed ratio from 40 to 80 ([*M*_0_]: 40 DIPG + 40 glycolide) and 120 ([*M*_0_]: 60 DIPG + 60 glycolide), respectively, while keeping the mol of initiator and catalyst constant.^1^H NMR (CDCl_3_, 400 MHz) for PDIPG–PGA **11** δ: 0.97–1.12 (m, 12H, 4CH_3_), 2.26–2.43 (br-s, 2H, 2CH), 4.56–4.93 (m, 4H, 2CH_2_), 4.93–5.09 (m, 2H, CH), 7.31–7.40 (m, 5H, 5xCH). ^13^C NMR (CDCl_3_, 100 MHz): δ 16.59–16.98, 18.39–18.76, 30.00–30.33, 60.40–61.03, 76.80, 166.27–167.02, 168.30–168.99. CHCl_3_ was fixed to 77.1 ppm. ATR-FTIR (ν_max_/cm^–1^): 2969, 2882 (CH); 1749 (C=O).

### Hydrolytic Degradation Studies of Copolymers

Time-dependent hydrolytic degradation studies of PDIBG–PGA **8** and PDIPG–PGA **11** copolymers were carried out at 37 °C, 100 rpm, and pH 7.4 in 3 replicates. 40 mg each of PDIPG–PGA and PDIBG–PGA copolymers were weighed into two separate falcons, and 5 mL each of PBS at pH 7.4 was added to the falcons and dispersed by vortex. The samples were then placed in an incubator at 37 °C and 100 rpm for 7, 14, 21, 28, 35, and 60 days. At the indicated time intervals, the samples taken from the incubator were centrifuged at 15,000 rpm for 15 min at 25 °C, and the supernatant was removed carefully with a pipet. The pellet was washed with 5 mL of distilled water, and it was centrifuged once under the same conditions. Then, the pellet was freeze-dried under a vacuum.

### Thin Film Formation of Copolymers

The thin films of the PDIPG–PGA **11**, PDIBG–PGA **8**, and PDIBG–PGA **8**-silica composites were obtained by the dip coating method, where the copolymer dissolved in THF at a copolymer concentration of 10 mg/mL. PDIBG–PGA **8**-silica composites were prepared by the addition of silica nanoparticles (SNP) from 0 to 30% (weight). The measurements of air–water and underwater contact angles were conducted by the Data Physics GmbH OCA-15EC Contact Angle Measuring System. The air contact angles of the surfaces were seized at three distinct locations for both the interface of the air-test liquids (θ_app_) with a maximum deviation of ±2°. The tilt angle (TA) experiment was conducted Data Physics GmbH OCA-15EC with a TA apparatus for 20 μL water and 5 μL hexadecane drops.^26^ For air contact angles, 5 μL of water (W), diiodomethane (DM), formamide (FA), α-bromonaphthalene (BN), and EG were dropped on the thin films. The surface tension of the test liquids was also determined by the pendant drop method. The surface free energy (SFE) values were calculated using the contact angle similar to the literature using the Owens-Wendt method.^27,28^ The surface roughness of the polymer films was examined using an atomic force microscope (Nanosurf Naio) in noncontact (wave) mode, scanning 10 μm × 10 μm areas under ambient conditions.^29^

## Results and Discussion

### Characterization of Monomers and Copolymers

l-DIBG **5** and l-DIPG **6** monomers were synthesized by self-condensation of l-2-hydroxy-4-methylpentanoic acid **3** or l-2-hydroxy-3-methylbutanoic acid **4** in toluene at reflux temperature in the presence of PTSA, respectively.^16,23^ Spectroscopic characterizations of l-2-hydroxy-4-methylpentanoic acid **3**, l-2-hydroxy-3-methylbutanoic acid **4**, l-DIBG **5**, and l-DIPG **6** were carried out by ATR-FTIR, ^1^H NMR, and ^13^C NMR techniques (Figures S3–S6, S14-S17, S22–S25).

**Scheme 1:**
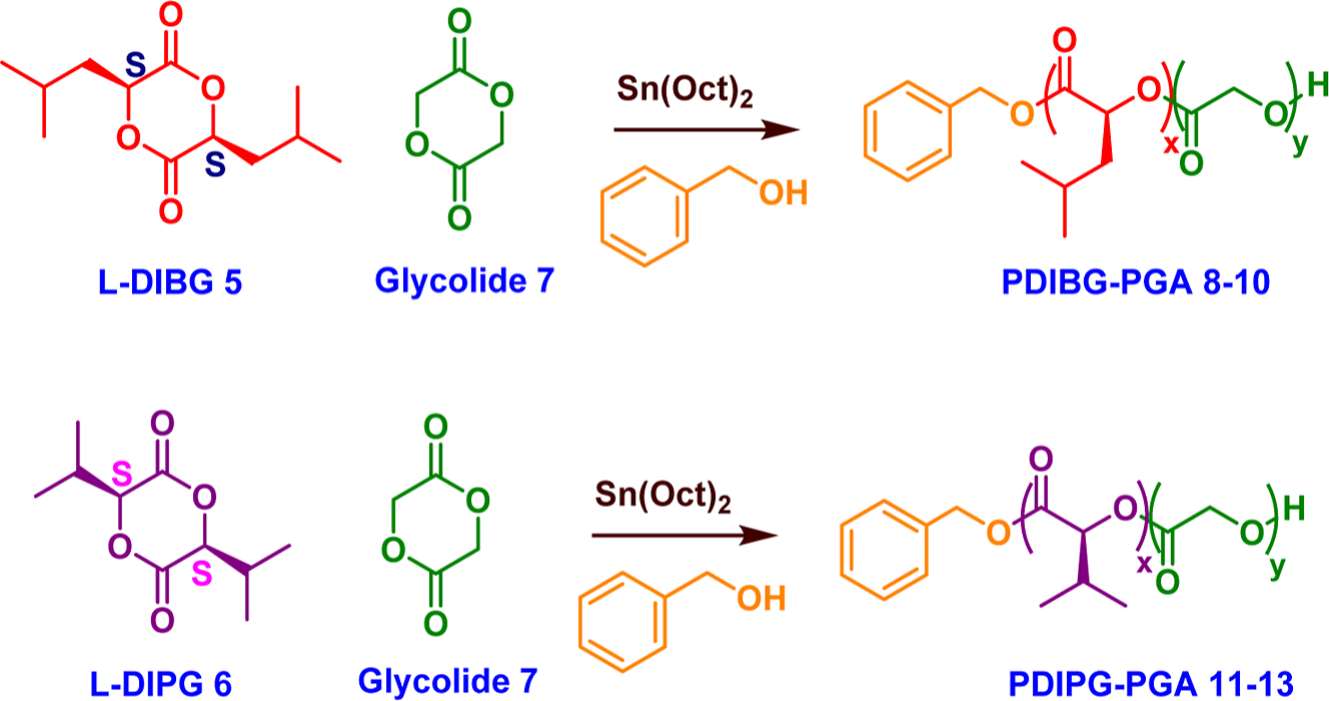
Synthesis of PDIBG–PGA **8–10** and PDIPG–PGA **11–13** Copolymers

**Figure 1:**
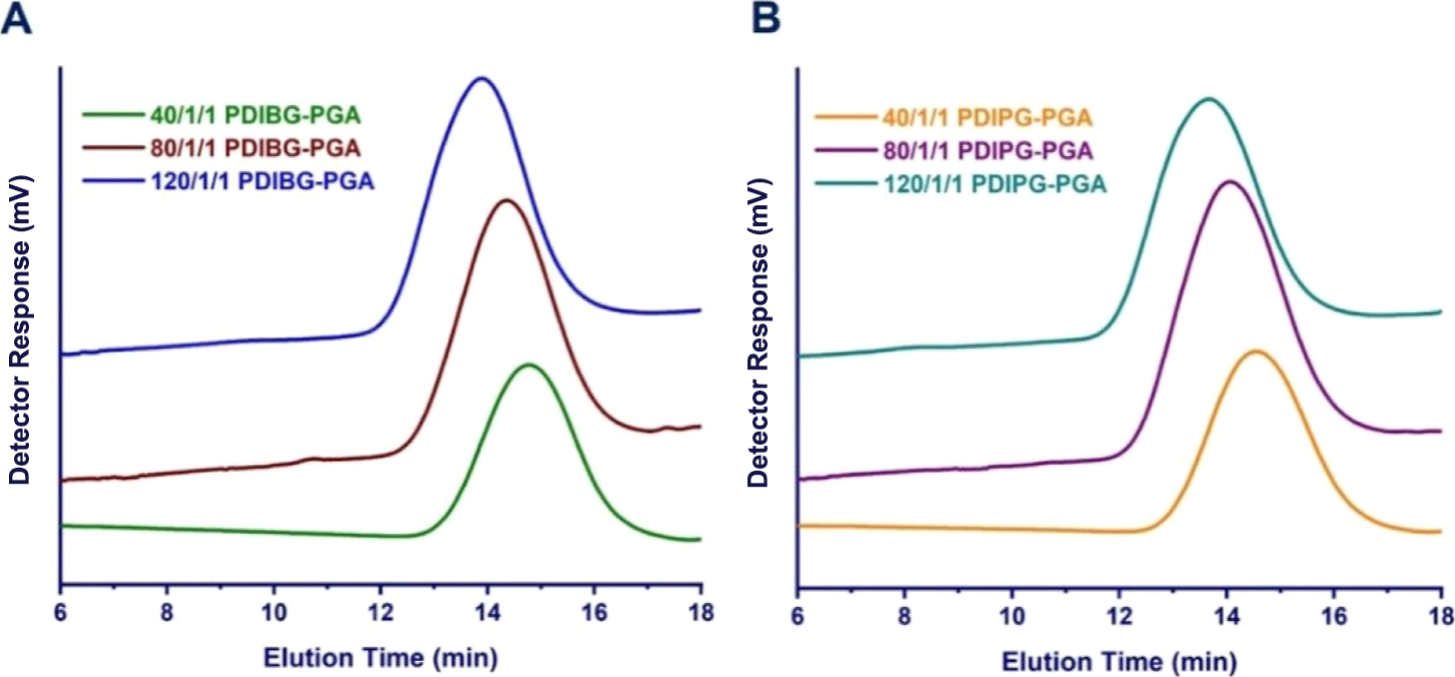
GPC chromatograms of PDIBG–PGA **8**–**10** (A) and PDIPG–PGA **11**–**13** copolymers (B).

**Figure 2:**
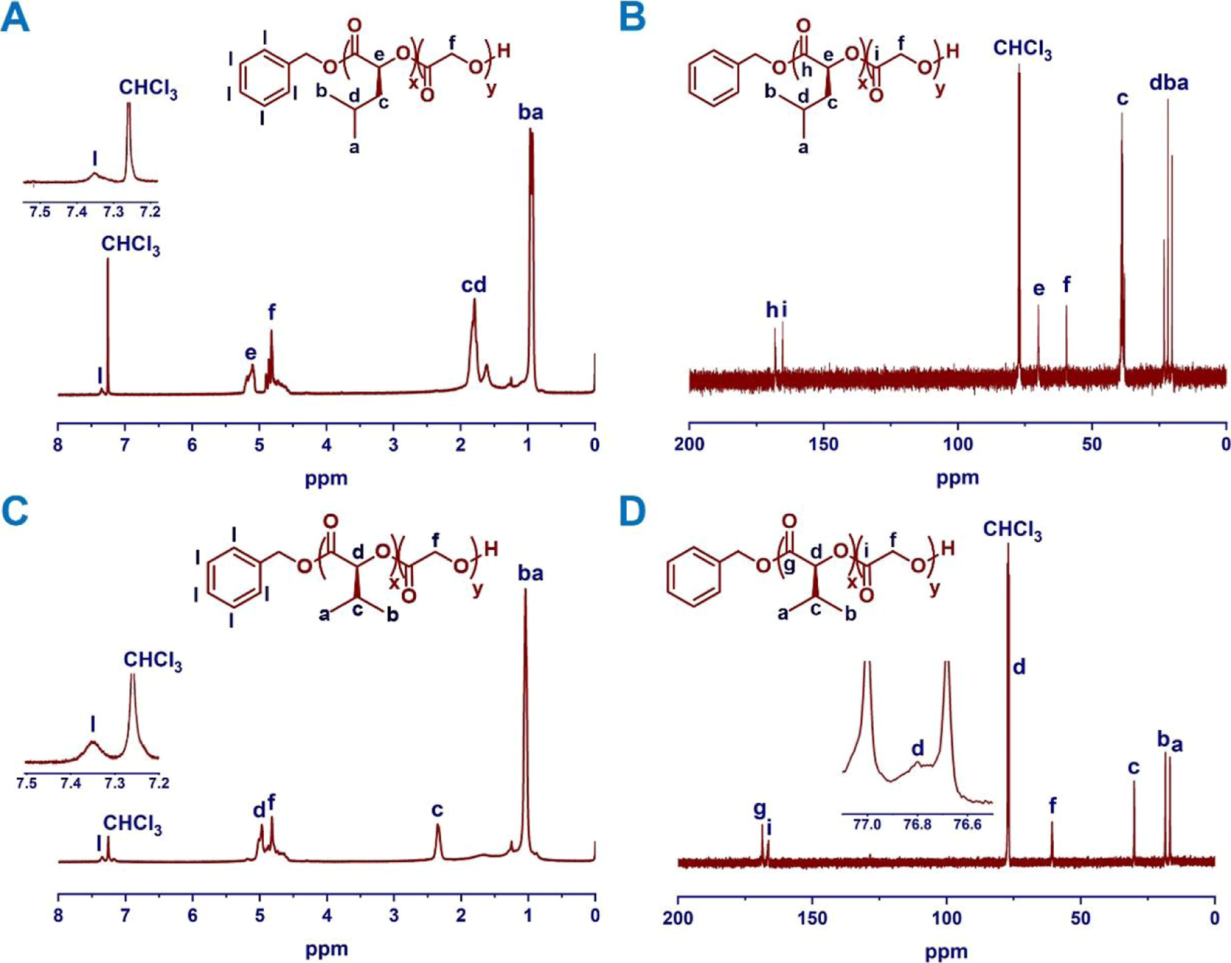
^1^H NMR (A,C) and ^13^C NMR (B,D) spectra of PDIBG–PGA **8** and PDIPG–PGA **11** copolymers in CHCl_3._.

PDIBG–PGA **8**–**10** and PDIPG–PGA **11**–**13** copolymers with various *M*_n_ values were synthesized by ring-opening copolymerization of l-DIBG **5** or l-DIPG **6** with glycolide **7** in the presence of benzyl alcohol as an initiator and Sn(Oct)_2_ as a catalyst (Scheme 1). *M*_n_ values of all copolymers **8**–**13** were close to their targeted molecular weights according to calculations of ^1^H NMR spectra (Table 1, Figure 2). They showed unimodal distributions according to GPC analyses (Figure 1) and high yields (up to 85%) (Table 1) based on gravimetric calculations.

**Table 1:** Characterization of PDIBG–PGA **8–10** and PDIPG–PGA **11–13** Copolymers

cop	ID	[*M*_0_]/[*I*_0_]/[C_0_]	*M*_w_^a^ (g/mol)	*M*_n_^a^ (g/mol)	*M*_n_^b^ (g/mol)	*M*_n_^c^ (g/mol)	exp. S.G./G. (%)^b^	*M*_w_/*M*_n_^a^	yield %^d^	inf. point^e^ (°C)	char yield %^e^
**PDIBG–PGA**	**8**	40/1/1	15,520	9560	8740	7000	53.1/46.9	1.62	78.1	298.2	3.0
	**9**	80/1/1	22,530	13,760	15,470	13,770	55.4/44.6	1.64	76.4	298.2	4.9
	**10**	120/1/1	34,090	20,850	19,800	20,660	55.4/44.6	1.64	67.0	297.8	3.9
**PDIPG–PGA**	**11**	40/1/1	18,280	10,670	7080	6330	54.2/45.8	1.71	81.0	310.4	5.1
	**12**	80/1/1	27,930	15,520	13,490	12,650	53.5/46.5	1.80	83.4	314.6	6.1
	**13**	120/1/1	39,950	23,360	18,850	18,980	58.7/41.3	1.71	85.0	317.1	3.3

a Determined by GPC.

b Calculated by ^1^H NMR.

c Theoretical molecular weight.

d Calculated by gravimetrically.

e Determined by TGA. S.G: substituted glycolide, G.:glycolide. Theoretical S.G./G. (%): 50/50. *M*_n_^b^ was calculated utilizing the aromatic protons of benzyl alcohol, and the methine proton of poly(substituted glycolide) and the methylene proton of polyglycolide.

As shown in Figure 1, all copolymers were obtained with monomodal peaks, and keeping the initiator ratio in the reaction medium constant and increasing the monomer (glycolide and l-DIBG or l-DIPG) feed ratios resulted in a corresponding increase in *M*_n_ of the copolymers determined by GPC (*M*_n_^GPC^ for 40:1:1 PDIBG–PGA **8**: 9560 g/mol vs for 120:1:1 PDIBG–PGA **10**: 20,850 g/mol; *M*_n_^GPC^ for 40:1:1 PDIPG–PGA **11**: 10,670 g/mol vs for 120:1:1 PDIPG–PGA **13**: 23,360 g/mol) (Table 1). However, the slightly higher PDI values (1.62–1.80) found for all copolymers are due to the fact that the polymerization reactions were carried out at high temperatures (180–190 °C) due to the high melting points of the l-DIBG (∼170 °C)^15,16^ and l-DIPG (∼160 °C)^15,23^ monomers. Although Sn(Oct)_2_ is often a preferred catalyst, it was reported in some studies that it may cause transesterification reactions at high temperatures, and PDI values were broadened due to uncontrolled side reactions occurring at high temperatures.^30−32^

Structural characterization of PDIBG–PGA **8** and PDIPG–PGA **11** copolymers was carried out by spectroscopic ^1^H NMR and ^13^C NMR techniques (Figure 2). The chemical shift of methine proton from 4.87 to 4.97 ppm for l-DIBG **5** (Figure S16) to 5.04–5.25 ppm for PDIBG–PGA copolymer **8** in the ^1^H NMR spectra, the chemical shift of methine carbon from 74.06 ppm for l-DIBG **5** (Figure S24) to 69.97 ppm for copolymer **8**, and the chemical shift of the carbonyl carbon from 167.33 ppm for l-DIBG **5** (Figure S24) to 167.73–168.23 ppm for copolymer **8** in the ^13^C NMR spectra confirmed the ROP (Figure 2A, 2B). Moreover, in the ^1^H NMR spectrum of the PDIBG–PGA **8** copolymer (Figure 2A), the ratio of the integral areas of a methine proton in PDIBG units and methylene protons in PGA units is 1:2, proving that the DIBG/Glycolide ratio in the copolymer is approximately 50:50 (Table 1). Similar results were also obtained in ^1^H NMR (Figure 2C) and ^13^C NMR (Figure 2D) analyses of PDIPG–PGA **11** and NMR analyses of PDIBG–PGA **9**, **10** and PDIPG–PGA **12, 13** copolymers (Figures S18–S21).

### Hydrolytic Degradation of Copolymers

**Figure 3:**
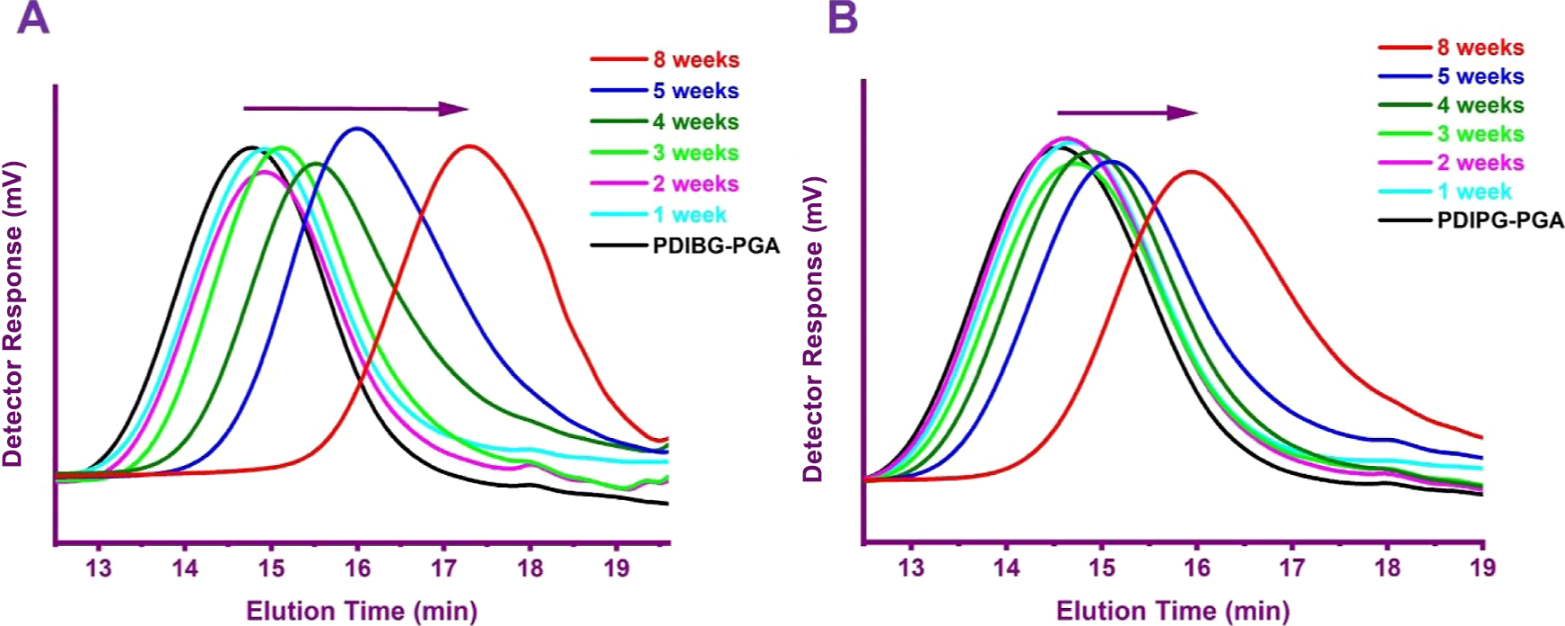
Degradation curves of PDIBG–PGA **8** (A), and PDIPG–PGA **11** (B), copolymers at different times.

Bulk degradation is dominant in polyesters such as PLA, PLGA, and PGA, and in this type of degradation, a nonlinear mass loss occurs with time.^33^ Hydrolytic degradation of polyesters such as PLA occurs by the diffusion of water into the amorphous regions of the polymer, the decrease in the molecular weight of the polymer by the breakage of ester bonds, and the formation of oligomers/monomers.^34^ Time-dependent (1, 2, 3, 4, 5, and 8 weeks) hydrolytic degradation behaviors of PDIBG–PGA **8** and PDIPG–PGA **11** copolymers in PBS buffer at 37 °C were evaluated by GPC analysis (Table 2, Figure 3). The degradation of PDIBG–PGA and PDIPG–PGA copolymers via mainly a bulk erosion mechanism showed that the degradation process consisted of two stages. First, *M*_n_ measured by GPC decreased slowly (1 and 2 weeks in Table 2) with little mass loss (data not shown), and then, the decrease of molecular weight increased (3–8 weeks in Table 2) with definite mass loss, namely, the molecular weight decreases of 88.3% and 76.5% and gravimetrically calculated mass losses of 36.7% and 12.3% were observed for PDIBG–PGA and PDIPG–PGA copolymers after 8 weeks of hydrolytic degradation, respectively. The mass loss was related to the formation of monomer and highly oligomers (data not shown).

**Table 2:** Time-Dependent Hydrolytic Degradation of PDIBG–PGA **8** and PDIPG–PGA **11** Copolymers^a^

	time (week)	*M*_W_^GPC^ (g/mol)	*M*_n_^GPC^ (g/mol)	PDI	*M*_n_^GPC^ % loss
PDIBG–PGA 8	0	15,520	9560	1.62	
	1	13,570 ± 276	8350 ± 97	1.63 ± 0.015	12.7
	2	13,310 ± 221	8070 ± 10	1.65 ± 0.027	15.6
	3	10,950 ± 174	6650 ± 32	1.65 ± 0.017	30.4
	4	7400 ± 108	4320 ± 76	1.71 ± 0.006	54.8
	5	4670 ± 59	2320 ± 56	2.01 ± 0.037	75.7
	8	1720 ± 56	1120 ± 35	1.54 ± 0.018	88.3
PDIPG–PGA 11	0	18,280	10,670	1.71	
	1	16,550 ± 104	9490 ± 180	1.74 ± 0.027	11.1
	2	17,160 ± 144	9380 ± 174	1.83 ± 0.026	12.1
	3	15,290 ± 180	8640 ± 350	1.77 ± 0.065	19.0
	4	13,430 ± 27	7370 ± 66	1.82 ± 0.013	30.9
	5	11,020 ± 262	6160 ± 193	1.79 ± 0.027	42.3
	8	4930 ± 166	2510 ± 75	1.97 ± 0.009	76.5

a The % *M*_n_ loss of PDIBG–PGA **8** and PDIPG–PGA **11** copolymers at different times (1, 2, 3, 4, 5, and 8 weeks) was calculated using the equation “(*M*_n,0_^GPC^ – *M*_n,t_^GPC^)/(*M*_n,0_^GPC^) × 100”.

PDIBG–PGA **8** was found to degrade faster than PDIPG–PGA **11** at all-time intervals tested (Figure 3, Table 2, *M*_n_^GPC^ loss at week 1:12.7% for PDIBG–PGA **8** vs 11.1% for PDIPG–PGA **11**; *M*_n_^GPC^ loss at week 2:15.6% for PDIBG–PGA **8** vs 12.1% for PDIPG–PGA **11**; *M*_n_^GPC^ loss at week 3:30.4% for PDIBG–PGA **8** vs 19.0% for PDIPG–PGA **11**; *M*_n_^GPC^ loss at week 4:54.8% for PDIBG–PGA **8** vs 30.9% for PDIPG–PGA **11**; *M*_n_^GPC^ loss at week 5:75.7% for PDIBG–PGA **8** vs 42.3% for PDIPG–PGA **11**; *M*_n_^GPC^ loss at week 8:88.3% for PDIBG–PGA **8** vs 76.5% for PDIPG–PGA **11**). The effect of *T*_g_ was found to be more dominant than hydrophobicity on the rate of hydrolytic degradation of the copolymers. The faster degradation of PDIBG–PGA **8** than PDIPG–PGA **11** can be attributed to a low *T*_g_ value (*T*_g_: 25.1 °C for PDIBG–PGA) below the hydrolytic degradation temperature (37 °C). At the degradation temperature, the rubbery state of PDIBG–PGA **8** chains accelerated the degradation of the copolymer by increasing the mobility of the polymer chains and allowing water to transfer more easily through the matrix.^20^ Conversely, the *T*_g_ value of 37.7 °C in PDIPG–PGA **11** copolymer, slightly above the degradation temperature, decreased the mobility of polymer chains and made the transfer of water molecules across the matrix more difficult, leading to slower degradation of the PDIPG–PGA **11** than the PDIBG–PGA **8**.^20,35^

### Thermal Analysis

**Figure 4:**
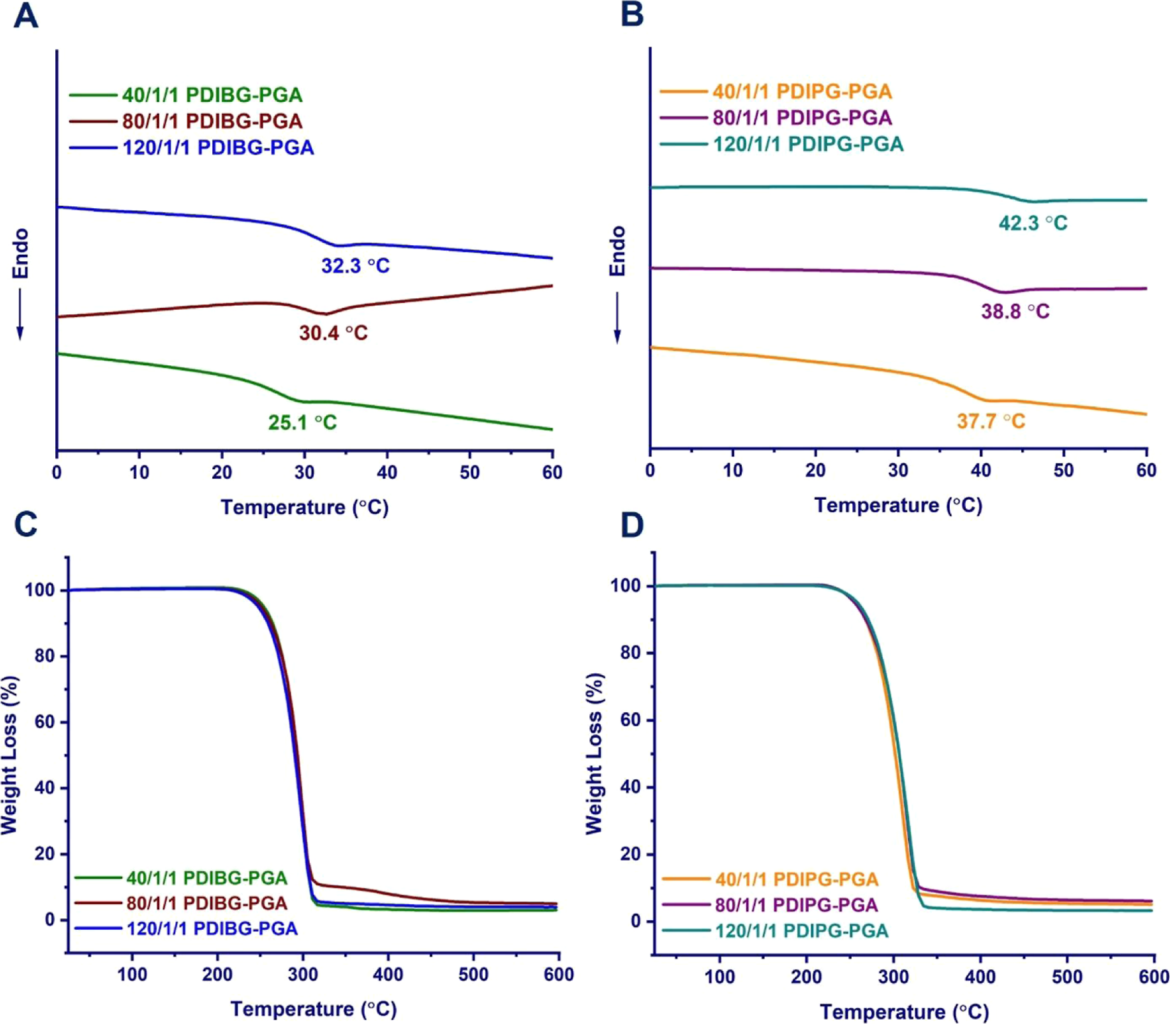
DSC (A,B) and TGA (C,D) curves of PDIBG–PGA **8**–**10** and PDIPG–PGA **11**–**13** copolymers.

DSC and TGA analyses were performed to evaluate the thermal behaviors of PDIBG–PGA **8**–**10** and PDIPG–PGA **11**–**13** copolymers. The influence of tertiary and secondary β-carbon atoms in alkyl side chains on *T*_g_ was clearly observed. The tertiary β-carbon atom of the alkyl side chains (−CH(CH_3_)_2_) in PDIPG–PGA **11**–**13** copolymers caused more inhibition of the rotation of the chains compared to PDIBG–PGA **8**–**10**, which contains a secondary β-carbon atom of the alkyl side chains (−CH_2_CH(CH_3_)_2_), leading to an increase in *T*_g_ (*T*_g_: 37.7 °C for PDIPG–PGA **11** vs 25.1 °C PDIBG–PGA **8**; *T*_g_: 38.8 °C for PDIPG–PGA **12** vs 30.4 °C PDIBG–PGA **9**; *T*_g_: 42.3 °C for PDIPG–PGA **13** vs 32.3 °C PDIBG–PGA **10**) (Figure 4A,B). Moreover, compared to the PDIBG–PGA copolymer containing an additional flexible –CH_2_ group in the alkyl side chain, the bulkier substituents in the PDIPG–PGA copolymer prevented the onset of segmental motion, resulting in a higher glass transition temperature. A similar situation was reported in the literature for PDIPG (*T*_g_: 41 °C^36^) and PDIBG (*T*_g_: 22 °C^19,24^) homopolymers. In addition, *T*_g_ was found to increase in both PDIBG–PGA and PDIPG–PGA copolymers in accordance with the molecular weight increase, as expected *T*_g_: 25.1 °C for PDIBG–PGA **8** (*M*_n_^GPC^: 9560 g/mol) vs *T*_g_: 30.4 °C for PDIBG–PGA **9** (*M*_n_^GPC^: 13,760 g/mol) vs *T*_g_: 32.3 °C for PDIBG–PGA **10** (*M*_n_^GPC^: 20,850 g/mol); *T*_g_: 37.7 °C for PDIPG–PGA **11** (*M*_n_^GPC^: 10,670 g/mol) vs *T*_g_: 38.8 °C for PDIPG–PGA **12** (*M*_n_^GPC^: 15,520 g/mol) vs *T*_g_: 42.3 °C for PDIPG–PGA **13** (*M*_n_^GPC^: 23,360 g/mol)). Moreover, no melting peak was observed for the copolymers in DSC analyses, indicating that all of the synthesized copolymers were amorphous. In the TGA analysis of all copolymers, a single mass loss was observed, as in the PLGA copolymers^37^ available in the literature (Figure 4C,D). PDIPG–PGA **11**–**13** copolymers were found to degrade at higher temperatures as their molecular weights increased (*M*_n_^GPC^: 10,670 g/mol and inflection point: 310.4 for **11** vs *M*_n_^GPC^: 15,520 g/mol and inflection point: 314.6 for **12** vs *M*_n_^GPC^: 23,360 g/mol and inflection point: 317.1 for **13**). However, somehow, the inflection points of the PDIBG–PGA **8**–**10** copolymers did not change with the increase in molecular weight (Table 1). In addition, in accordance with the hydrolytic degradation data determined by GPC analyses, it was also found that PDIBG–PGA **8**–**10** degraded faster than PDIPG–PGA **11**–**13** in TGA analyses.

### Surface Characterization of PDIPG–PGA and PDIBG–PGA Thin Films

The air and underwater contact angle results of thin films of PDIPG–PGA **11** and PDIBG–PGA **8** were collected in Table 3. It can be seen that the copolymer surfaces have a close hydrophilic/hydrophobic boundary of the water contact angle. As a result, it can be stated that both thin films demonstrate hydrophobic properties. Furthermore, the underwater hexadecane contact angle outcomes reinforce the findings of water contact angle measurements conducted in the air (Table 3) because the necessity of hydrophilic groups for achieving underwater superoleophobicity is an essential condition that cannot be overlooked.^27,38^ The SFE values of the thin film were also calculated with the Owens-Wendt method, as given in Table 3. The similarity in SFE values between both surfaces is apparent. Nevertheless, the PDIBG–PGA **8** surface exhibits a higher polar component of SFE compared to that of the PDIPG–PGA **11** surface.

**Table 3:** Air and Underwater Contact Angle and SFE Results on the Thin Films^a^

sample	contact angle (^o^)					surface free energy (mj/m^2^)		
	θ_app_^W^	θ_app_^DM^	θ_app_^FA^	θ_app_^EG^	θ_und_^H^	γ_S_^d^	γ_*S*_^*p*^	γ_S_^Tot^
**PDIPG–PGA11**	86.7	53.8	81.5	80.6	48	29.1	3.5	32.6
**PDIBG–PGA 8**	84.9	53.9	71.7	78.6	75	28.3	4.6	32.9

a Water (W), diiodomethane (DM), formamide (FA), ethylene glycol (EG), hexadecane (H).

The water contact angles of PDIPG and PDIBG thin films were reported to be 46° and 77°, respectively.^27,39^ Upon incorporation of PGA into these polymers, the water contact angles increased by 40° for PDIPG–PGA **11** and 8° for PDIBG–PGA **8**, respectively (Table 3). In terms of underwater hexadecane contact angles, the PDIBG–PGA **8** copolymer exhibited an angle 27° higher than that of the PDIPG–PGA **11** copolymer. A heightened polar component tends to enhance water interaction with the surface,^40^ consequently elevating the oil contact angle.^27,38^ PDIBG–PGA **8** exhibits stronger interactions with water molecules due to its higher proportion of polar components in the SFE, leading to a faster hydrolytic degradation rate. In contrast, PDIPG–PGA **11** demonstrates slower degradation because of its lower polar energy component and higher dispersive energy component, which provides resistance to water penetration (Tables 2 and 3). This highlights that the balance between the polar and dispersive components of the SFE is a key factor in determining the degradation rate of the polymer.

**Figure 5:**
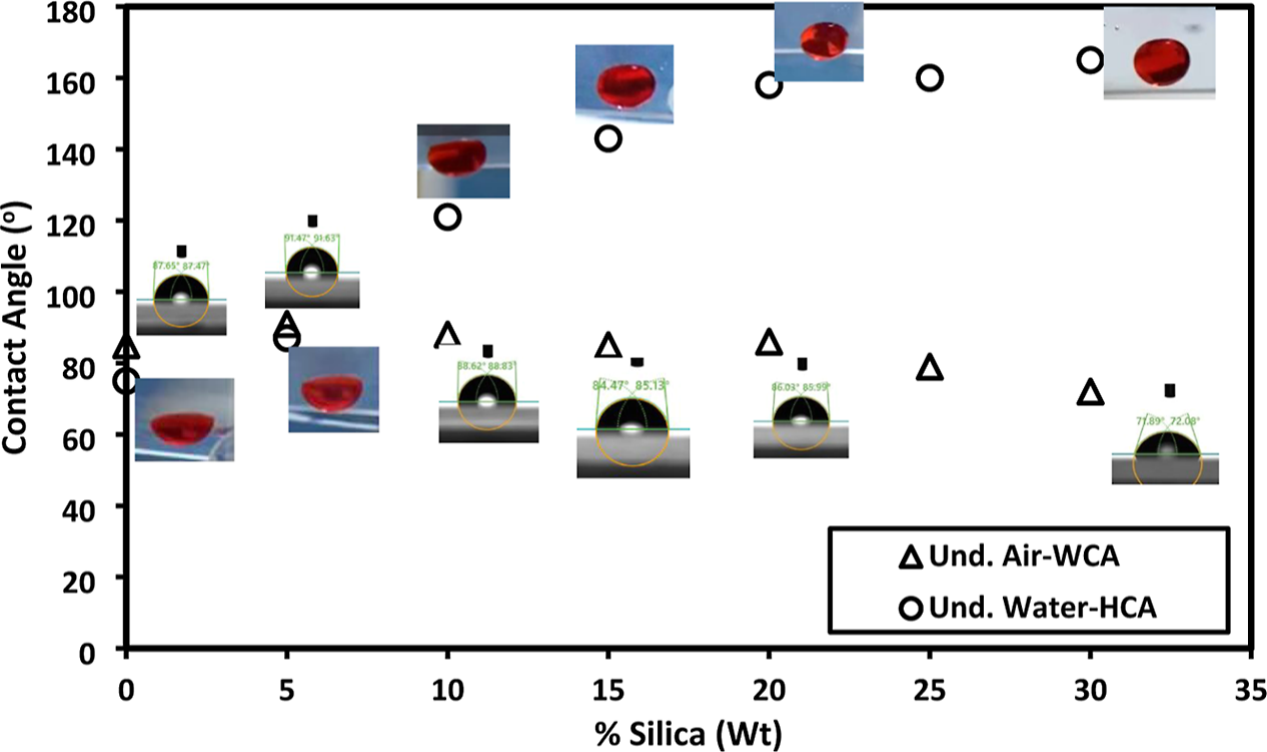
Under-air water (Δ) and under water hexadecane contact angle (o) results and its profile images by increasing the silica nanoparticles into PDIBG–PGA **8**.

The PDIBG–PGA **8** copolymer can rapidly gain water–oil repellent properties by the addition of hydrophilic particles. To enhance underwater superoleophobicity, especially in the case of the PDIBG–PGA **8** copolymer rather than PDIPG–PGA **11** copolymer due to the above reasons, we modified the copolymer composite surfaces, leveraging its higher θ_und_^H^ and γ_S_^p^ values, as demonstrated in Table 3. Thus, we incorporated hydrophilic silica nanoparticles into PDIBG–PGA **8** to augment the surface roughness. As the quantity of silica nanoparticles increases, the water contact angle in the air environment fluctuates between 85° and 72°, while the underwater hexadecane contact angle exhibits an increase from 75° to 165°, as given in Figure 5 and Supporting Information Video S1, attributed to the amphiphilic nature of the composite surfaces.

The hydrophobic part resulting from the isobutyl groups in the polymeric structure of PDIBG–PGA **8** and the hydrophilic parts originating from silica nanoparticles provide the composite coating with amphiphilic properties.^27^ The increased hydrophilic feature provides repellency to the surface against water penetration into the roughnesses under water and repels oil drops (Figure 5), aligning with the underwater Cassie state model.^41,42^ However, this hydrophilic attribute does not facilitate water penetration between the roughness in the air environment nor elevate the water contact angle to the superhydrophilic level due to the surface’s amphiphilic nature, attributed to hydrophobic regions.^27^ It can be concluded that the water repellency of hydrophobic regions under water is insufficient due to dense water molecules. The surface roughness parameter results (average and root mean square roughness) and the AFM images were collected in Table 4. The 2D topographic and 3D mapping illustrated an elevation in the silica content across the surfaces (Table 4). In particular, with silica content reaching 20% (wt), noticeable reduction in surface gaps was observed, with the initiation of a double-scale roughness.

**Table 4:**
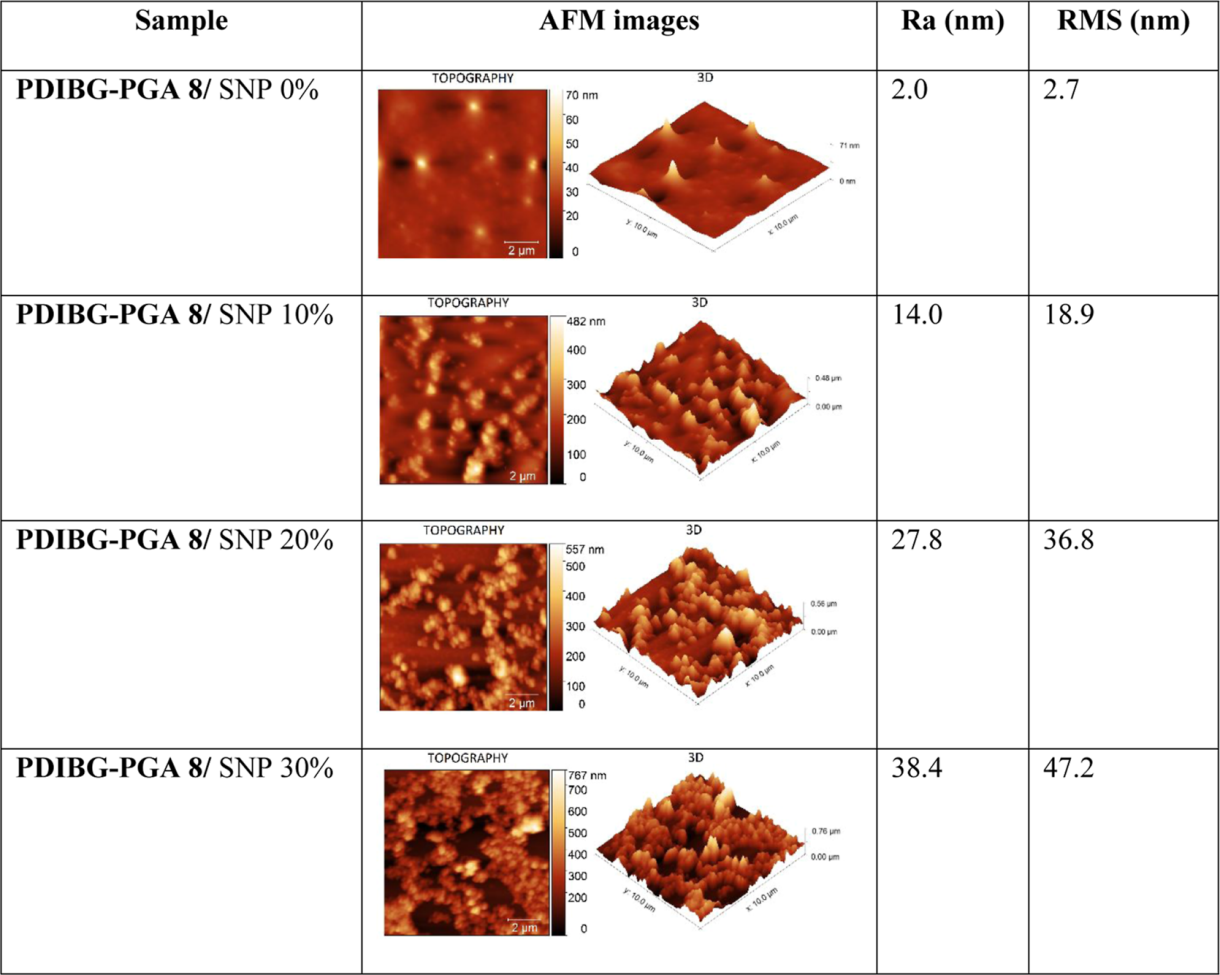
AFM Images and the Roughness Parameters of the Silica Incorporating Surfaces^a^

a Ra: average roughness; RMS: root mean square roughness.

The absence of silica within the composite structure, the smooth surface was obtained with RMS value of at 2.7 nm. A noticeable elevation in the RMS value became apparent with increasing silica content, peaking at 47.2 nm once the silica content reached 30% (Table 4). Correspondingly, AFM images reveal a hierarchical structuring concurrent with an increment in the RMS value increment.

**Figure 6:**
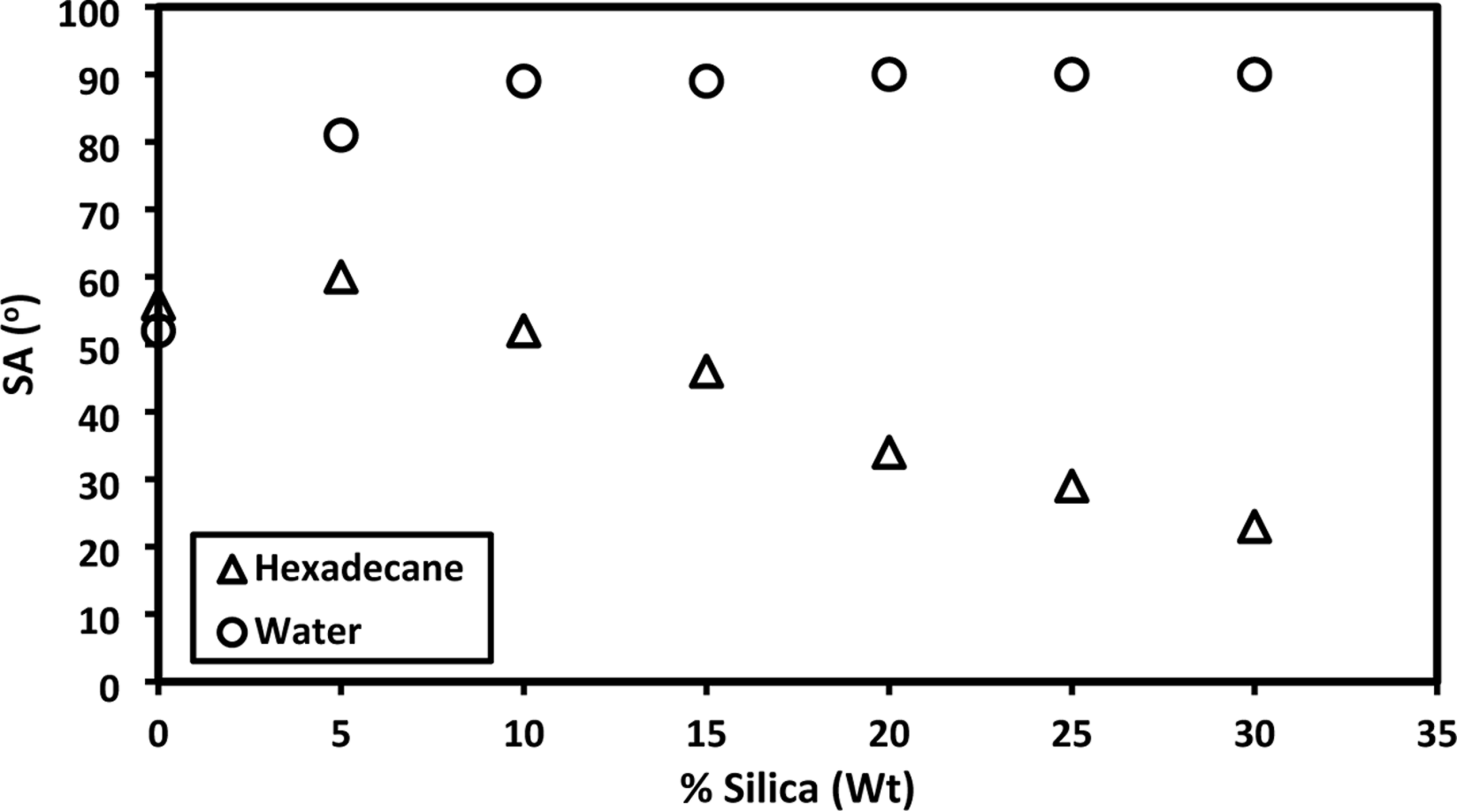
SA results of water and hexadecane drop depending on the silica content in PDIBG–PGA silica composites.

SA results of water and hexadecane drops were also collected in Tables 5 and 6, respectively. The SA data were obtained by recording the initial moment at which a liquid droplet of specified weight commenced sliding down the inclined plate.^43−45^ Nevertheless, although the droplet initiates sliding at a particular angle (SA value) on the surface, it may get stuck on the surface and not be able to be completely separated from the surface due to heterogeneity or roughness on the surface. Table 5 illustrates the variation in SA and contact angle hysteresis (CAH) as the silica content increases for a 20 μL water drop. The water droplet remains affixed to the surface at 90° inclination across all surfaces, except the smooth surface, which has an RMS value of 2.7 nm. Similarly, SA values stabilized at 90° after 10% silica content (Figure 6). When the CAH values of the water droplet are observed at a consistent TA of 30°, it becomes evident that the CAH values fluctuate within the range of 16° to 50° (Table 5).

**Table 5:**
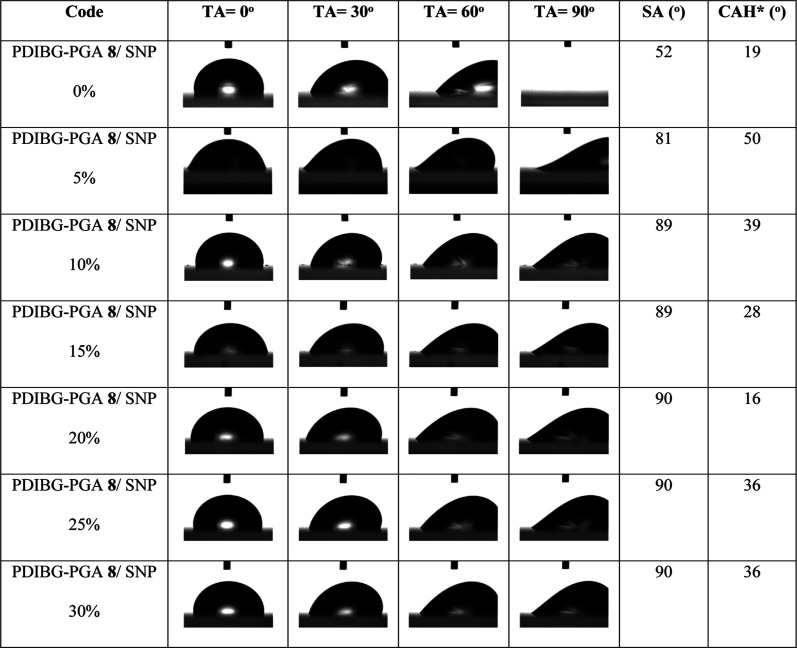
Water Drops Profile Images, SA, and CAH Results of the Composite Films at Different TA Angles Depending on the Silica Increase in PDIBG–PGA Silica Composites

a SA: sliding angle. CAH values of water drops were measured at the tilt angle (TA) values at 30°.

**Table 6:**
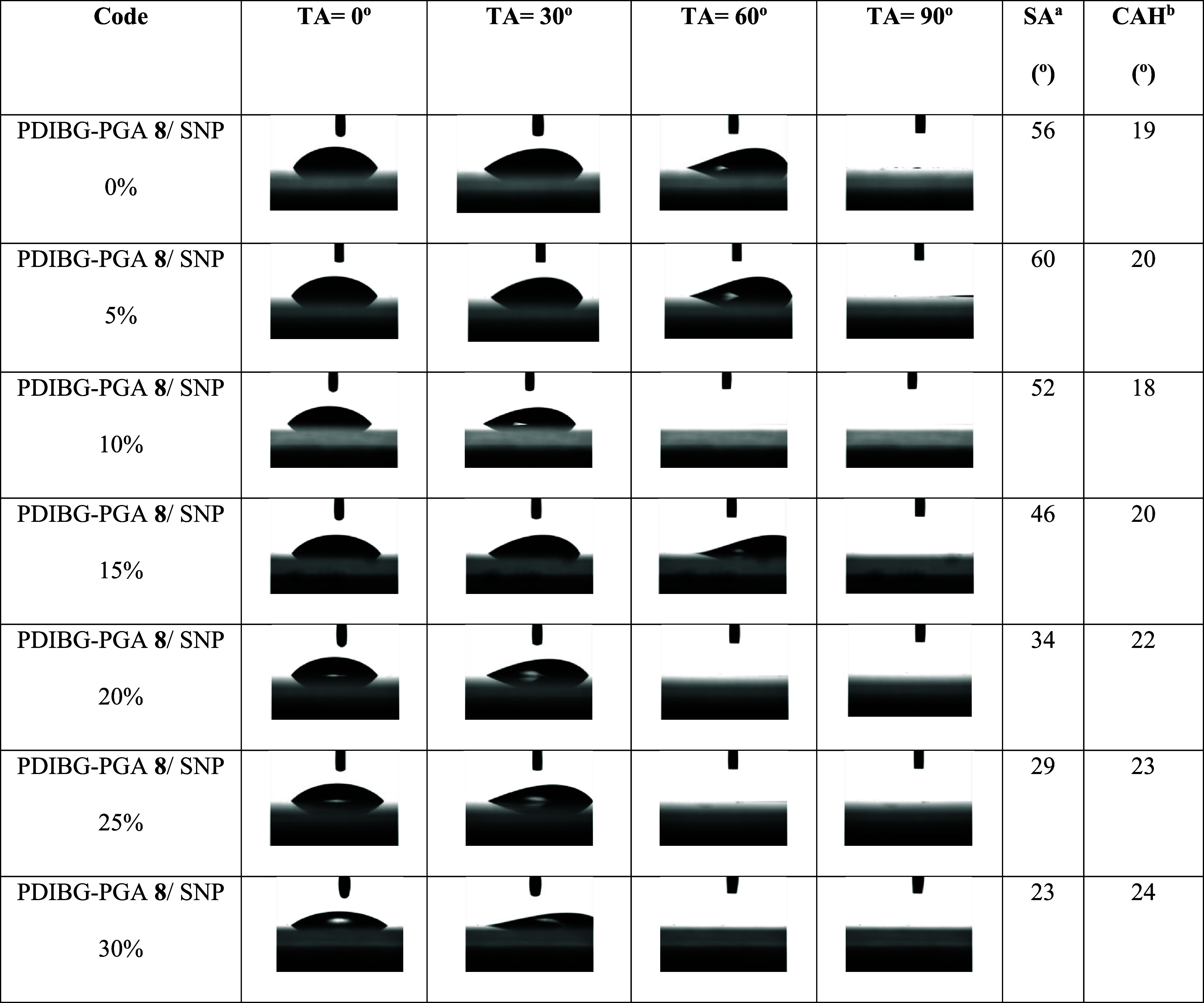
Hexadecane Drops Profile Images, SA, and CAH Results of the Composite Films at Different TA Angles Depending on the Silica Increase in PDIBG–PGA Silica Composites

a SA: sliding angle. CAH values of hexadecane drops were measured at the TA values at 20°.

Conversely, it is observed that the SA values of hexadecane liquid exhibit a linear decrease as roughness increases, as given in Table 6 and Figure 6. In Table 6, it is evident that as roughness increases, 5 μL of hexadecane liquid leaves the surface at lower SA values at tilt angles ranging from 0° to 90°. Additionally, the CAH value of hexadecane liquid remains relatively consistent within a narrow range of 18 to 24° at a fixed TA of 20°.

## Conclusions

In this study, a series of PDIPG–PGA and PDIBG–PGA copolymers of various molecular weights (*M*_n_^NMR^: between 7080 and 19,800 g/mol) with degradable properties were synthesized and characterized by spectroscopic (NMR, ATR-FTIR), chromatographic (GPC), and thermal (DSC, TGA) methods in order to develop new alternative biomaterials to PLGA copolymers. DSC analysis revealed that PDIBG–PGA copolymers containing secondary β-carbon atoms in alkyl side chains (−CH_2_CH(CH_3_)_2_) had lower T_g_ than PDIPG–PGA copolymers containing tertiary β-carbon atoms in alkyl side chains (CH(CH_3_)_2_) (for instance, T_g_: 25.1 °C for PDIBG–PGA vs T_g_: 37.7 °C for PDIPG–PGA). In time-dependent (1, 2, 3, 4, 5, and 8 weeks) hydrolytic degradation experiments of the copolymers in PBS buffer at 37 °C, PDIBG–PGA **8** (*T*_g_: 25.1 °C) copolymers with a *T*_g_ below the degradation temperature degraded faster than PDIPG–PGA **11** (*T*_g_: 37.7 °C) copolymers at all time intervals tested (*M*_n_^GPC^ loss: 88.3% for PDIBG–PGA **8** vs 76.5% for PDIPG–PGA **11** at week 8). In addition, in accordance with the hydrolytic degradation results, TGA analysis revealed that PDIBG–PGA copolymers degraded faster than PDIPG–PGA copolymers. The synthesized novel PDIBG–PGA and PDIPG–PGA copolymers can be considered as alternative materials with degradable properties that can be used in biomedical applications. Especially, the controlled degradation behavior of these copolymers allows their potential use as drug delivery systems. The PDIBG–PGA copolymer, which exhibited greater underwater oleophobic properties compared with the PDIPG–PGA copolymer, demonstrated underwater superoleophobicity with the introduction of hydrophilic silica, enhancing surface roughness and hydrophilicity. Despite the increased RMS value, the surfaces retained moderate dynamic oleophobicity in air while maintaining transparency. These modifications expand the potential applications of surfaces in critical fields such as self-cleaning, bioadhesion prevention, marine antifouling, and microfluidic technology. This study serves as a foundation for diverse applications, leveraging its underwater superoleophobic properties and low shear angle values due to increased silica content. The elevated surface roughness and the presence of low CAH values offer a distinct advantage in microfluidic systems by facilitating efficient fluid movement across surfaces. Additionally, their degradable nature and tunable degradation behavior make them ideal candidates for industrial applications, such as controlled drug delivery systems or ecofriendly food packaging. The increased roughness also functions as a protective barrier against biological contaminants in underwater environments, effectively preventing oil adhesion. This minimizes the contact area between the surface and oil droplets, improving self-cleaning capabilities, antifouling performance, and contamination resistance. As a result, this mechanism significantly reduces biological fouling, ensuring an enhanced surface functionality under challenging conditions.

## Supporting Information Available

ATR-FTIR, ^1^H NMR, and ^13^C NMR spectra (PDF)
Effect of increasing silica content (0–30 wt %) on underwater hexadecane contact angle (MP4)
