# Supplementary material for: Synthesis, Hydrolytic Degradation Behavior, and Surface Properties of Poly(alkyl glycolide)-Polyglycolide Copolymers
Source: ACS Omega. 2025 Feb 20;10(8):8499–511. doi: 10.1021/acsomega.4c10768 (PMC11886707; doi:10.1021/acsomega.4c10768)
Supplement: Supplementary file 1 — ao4c10768_si_001.pdf [file ao4c10768_si_001.pdf]

Synthesis, Hydrolytic Degradation Behavior and Surface Properties of Poly(Alkyl Glycolide)-Polyglycolide Copolymers

Mehtap Cantürk Bamyacı[a]*, Duygu Çetin*[a]*, Candan Cengiz*[b]*, Sema Nur Belen*[c]*, Olcay Mert*[a,d]*, Ugur Cengiz*[b,e] *and Serap Mert* [a,f,g]*

[a]*Department of Polymer Science and Technology, Kocaeli University, 41001, Kocaeli, Türkiye*

[b]*AFC Green Technologies R&D, Canakkale Technopark, Sarıcaeli, 17100, Çanakkale, Turkiye*

[c]*Department of Energy Resources and Management, Faculty of Engineering, Çanakkale Onsekiz Mart University, 17100, Çanakkale, Türkiye*

[d]*Department of Chemistry, Kocaeli University, 41001, Kocaeli, Türkiye*

[e]*Surface Science Research Laboratory, Department of Chemical Engineering, Faculty of Engineering, Çanakkale Onsekiz Mart University, Çanakkale, Türkiye*

[f]*Center for Stem Cell and Gene Therapies Res. and Pract., Kocaeli University, 41001, Kocaeli, Türkiye*

[g]*Department of Chemistry and Chemical Processing Tech., Kocaeli University, 41140, Kocaeli, Türkiye*

* E-mail: serap.mert@kocaeli.edu.tr

General protocol for α-hydroxy acid synthesis

The synthesis of L-2-hydroxy-4-methylpentanoic acid (IBHA) **3** and L-2-hydroxy-3-methylbutanoic acid (IPHA) **4** hydroxy acids was carried out by diazotization of L-leucine **1** and L-valine **2** in the presence of H_2SO_4 and aqueous sodium nitrite solution, respectively, using the method known in the literatüre.[1-3]

General protocol for monomer synthesis

L-3,6-diisobutyl-1,4-dioxane-2,5-dione (L-DIBG) **5** and L-3,6-diisopropyl-1,4-dioxane-2,5-dione (L-DIPG) **6** monomers were prepared by condensation of L-2-hydroxy-4-methylpentanoic acid **3** and L-2-hydroxy-3-methylbutanoic acid **4** in toluene in the presence of p-toluene sulfonic acid monohydrate respectively, using the literature known method.[2,3]

Crystallization of Glycolide

2.5 g of glycolide **7** monomer was dissolved in 20 mL of dry tetrahydrofuran at 35 °C, and 20 mL of dry toluene was added dropwise to the solution at 40 °C until crystals started to form. The crystals formed were kept overnight under nitrogen gas, and then, at -22 °C for ~30 min to enhance crystallization, and separated from the filtrate by decantation (89% yield).[4]

Preparation of Phosphate Buffer Solution (PBS) (pH 7.4)

0.80 g NaCl, 0.02 g KCl, 0.14 g Na_2HPO_4, 0.024 g KH_2PO_4 were dissolved in 100 mL distilled water to obtain a homogeneous mixture. The solution with a pH value of 7.4 was filtered using filter paper and used in hydrolytic degradation experiments.

ATR-FTIR Spectra

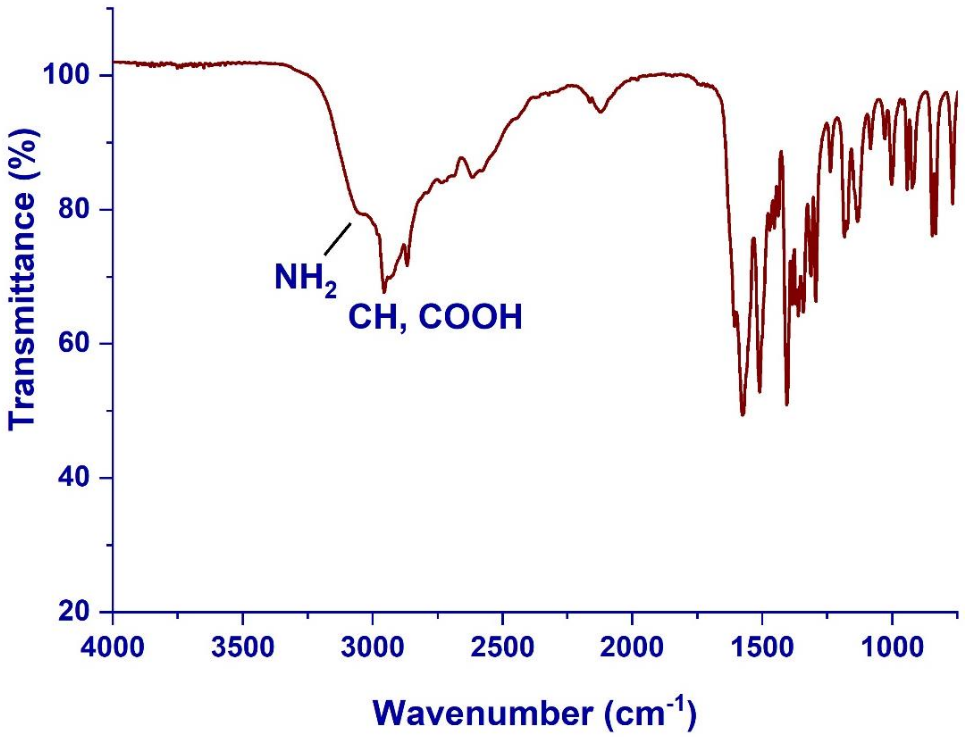

Figure S1. ATR-FTIR spectrum of L-Leucine **1**

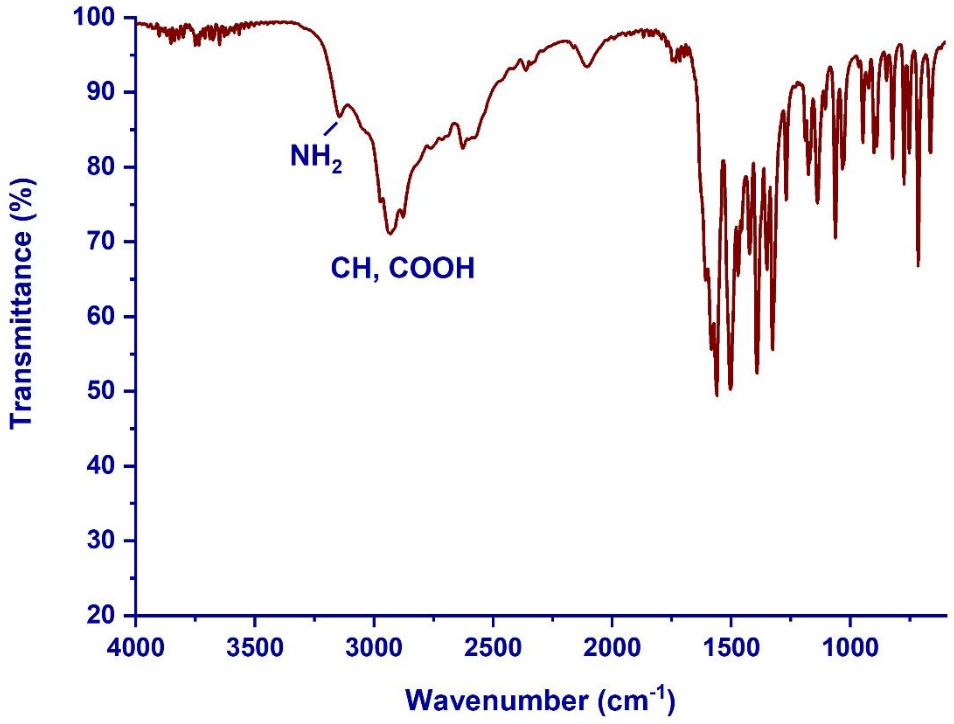

Figure S2. ATR-FTIR spectrum of L-Valine **2**

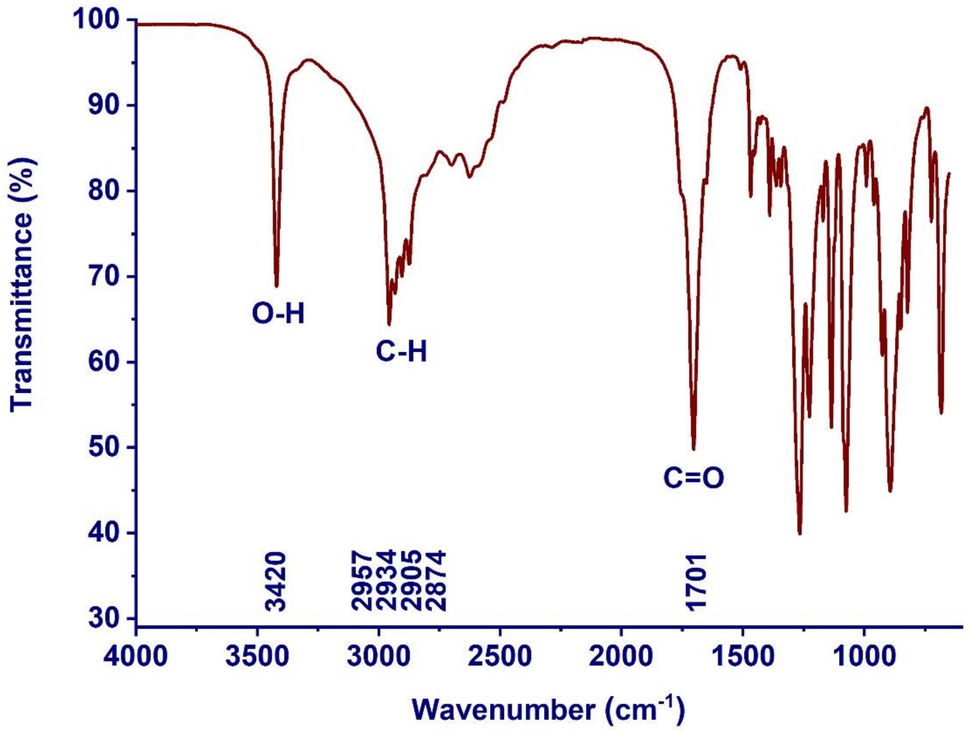

Figure S3. ATR-FTIR spectrum of L-2-hydroxy-4-methylpentanoic acid (IBHA) **3**

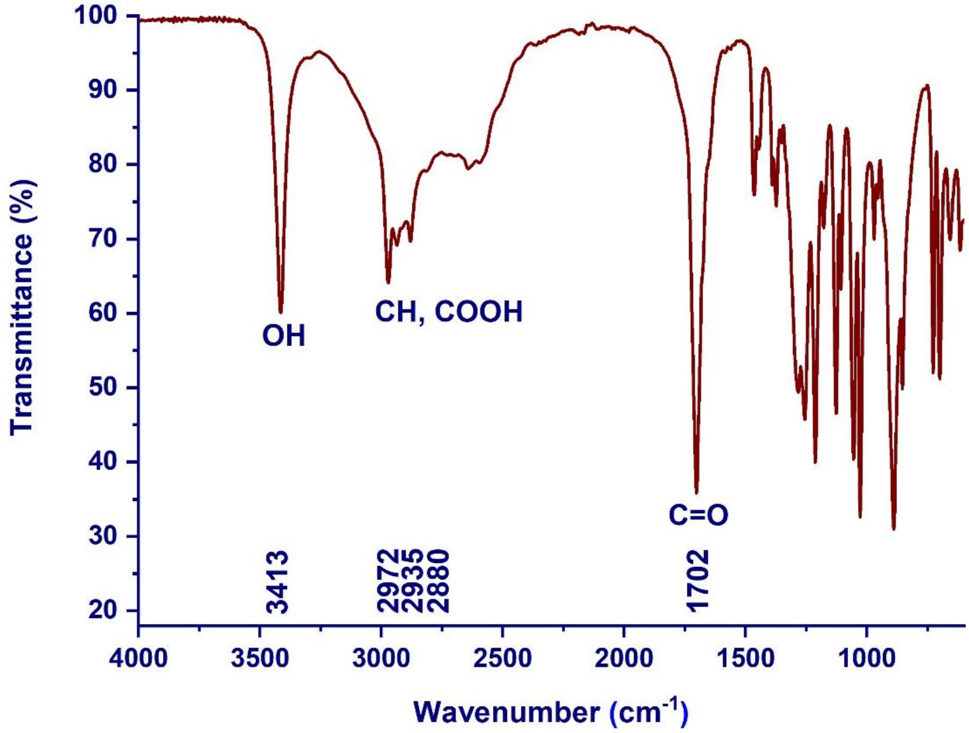

Figure S4. ATR-FTIR spectrum of L-2-hydroxy-3-methylbutanoic acid (IPHA) **4**

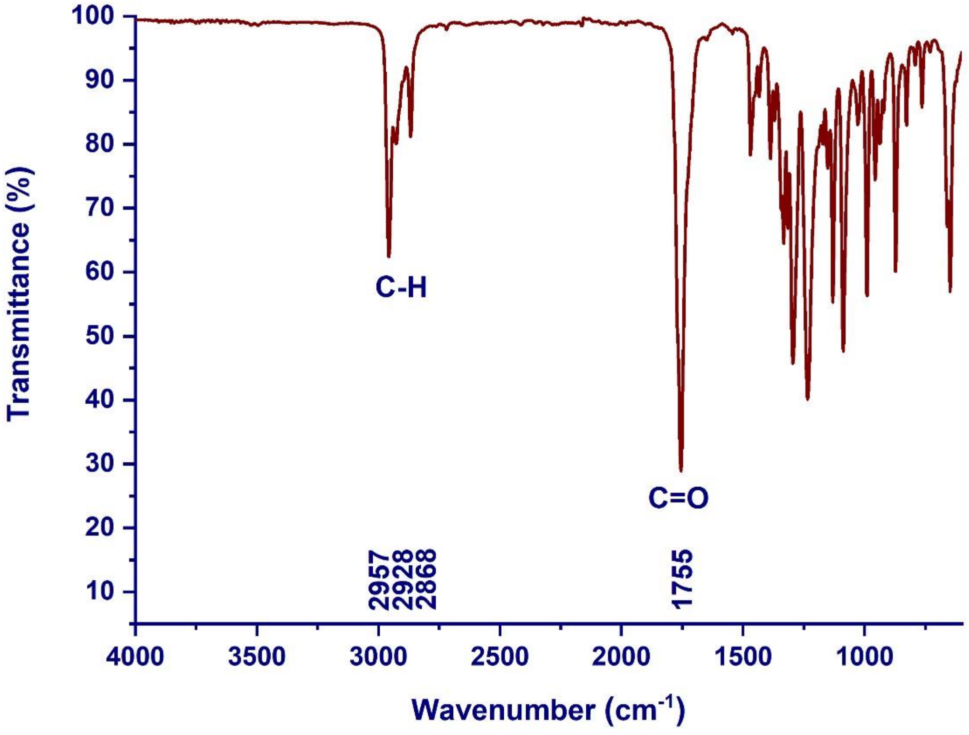

Figure S5. ATR-FTIR spectrum of L-3,6-diisobutyl-1,4-dioxane-2,5-dione (L-DIBG) **5**

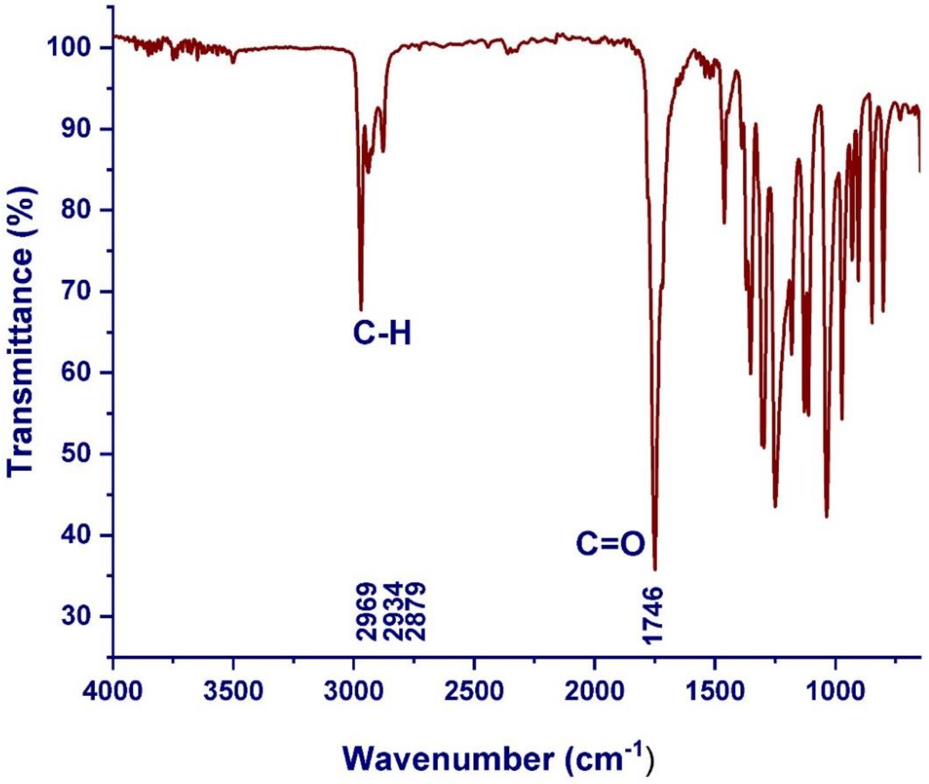

Figure S6. ATR-FTIR spectrum of L-3,6-diisopropyl-1,4-dioxane-2,5-dione (L-DIPG) **6**

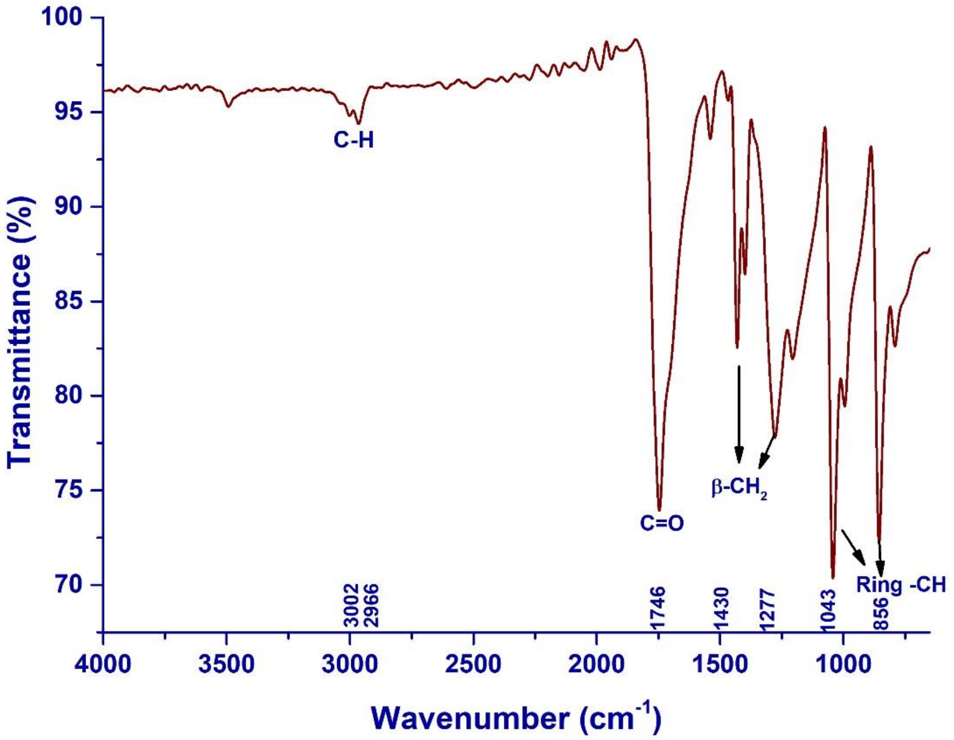

Figure S7. ATR-FTIR spectrum of glycolide **7**

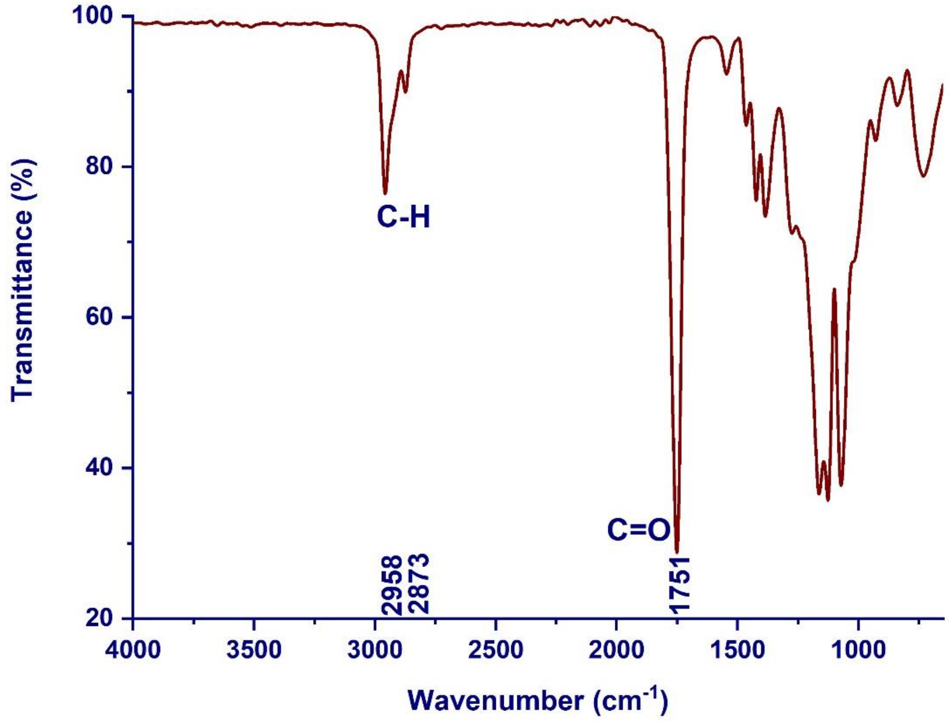

Figure S8. ATR-FTIR spectrum of PDIBG-PGA **8** copolymer

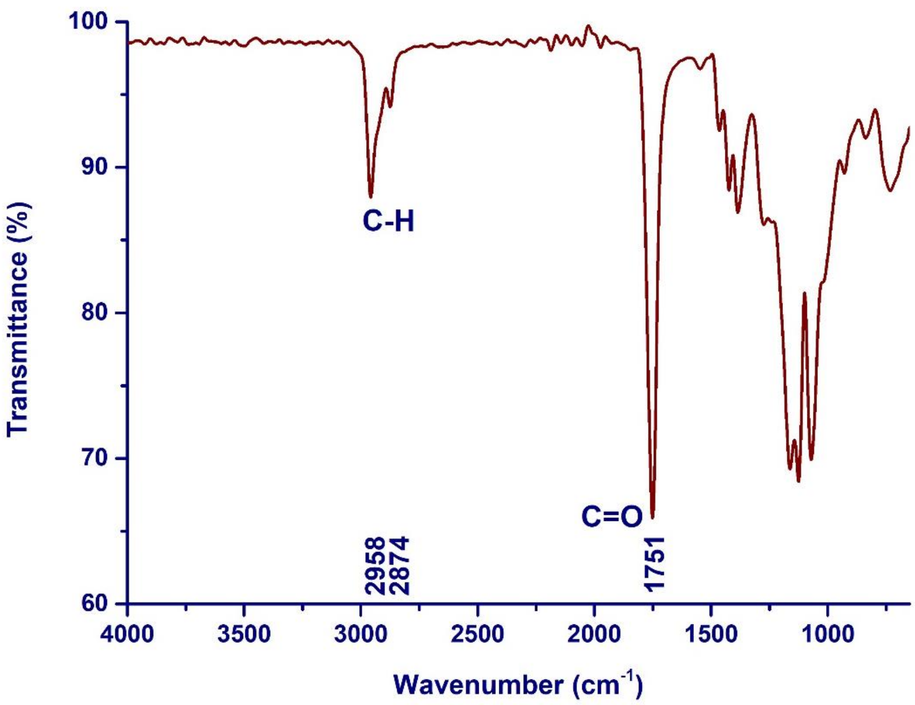

Figure S9. ATR-FTIR spectrum of PDIBG-PGA **9** copolymer

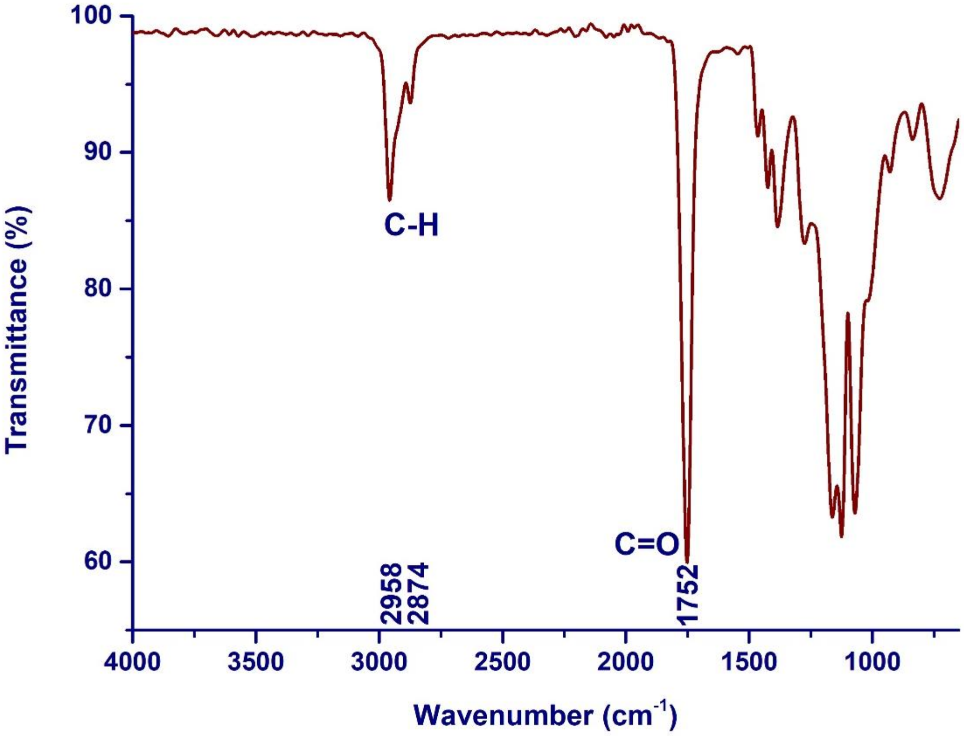

Figure S10. ATR-FTIR spectrum of PDIBG-PGA **10** copolymer

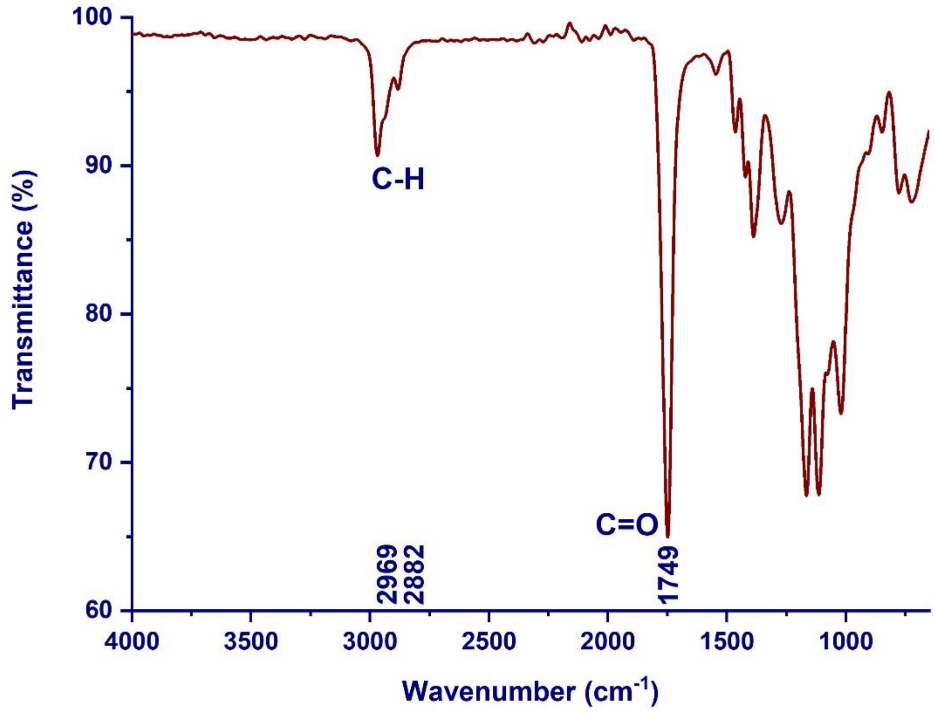

Figure S11. ATR-FTIR spectrum of PDIPG-PGA **11** copolymer

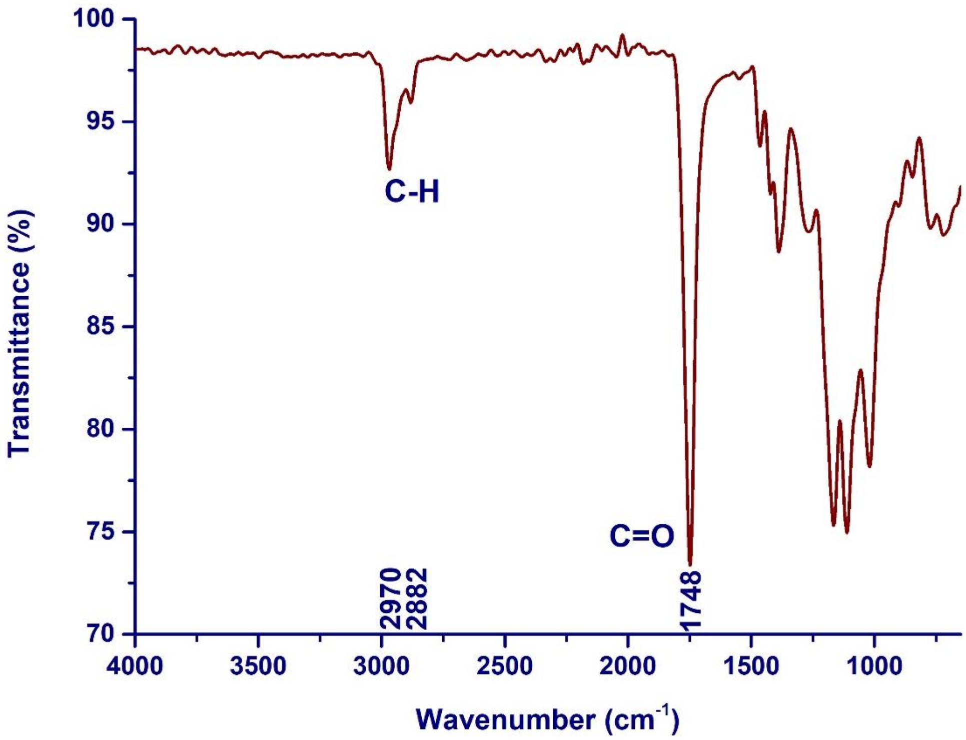

Figure S12. ATR-FTIR spectrum of PDIPG-PGA **12** copolymer

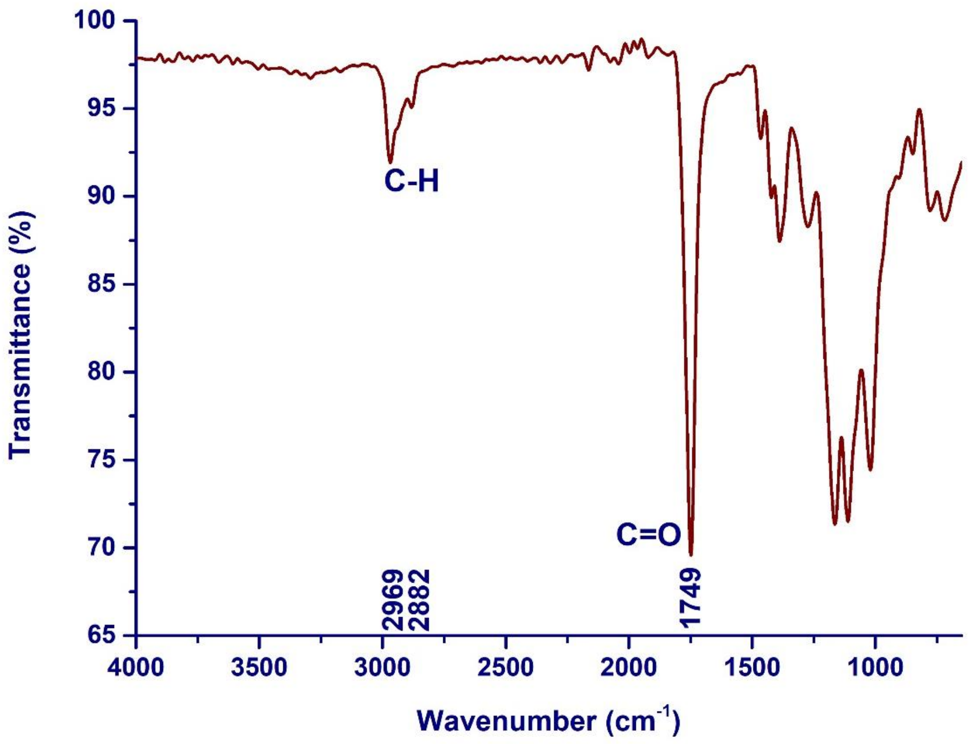

Figure S13. ATR-FTIR spectrum of PDIPG-PGA **13** copolymer

^{1}H-NMR Spectra

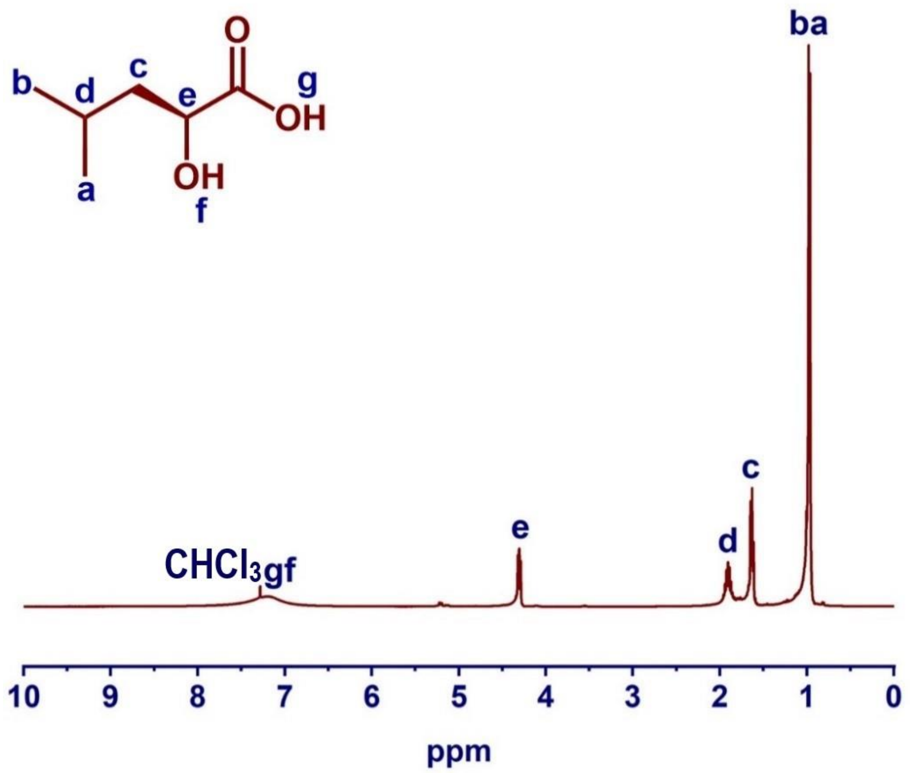

Figure S14. ^{1}H-NMR spectrum of L-2-hydroxy-4-methylpentanoic acid (IBHA) **3**

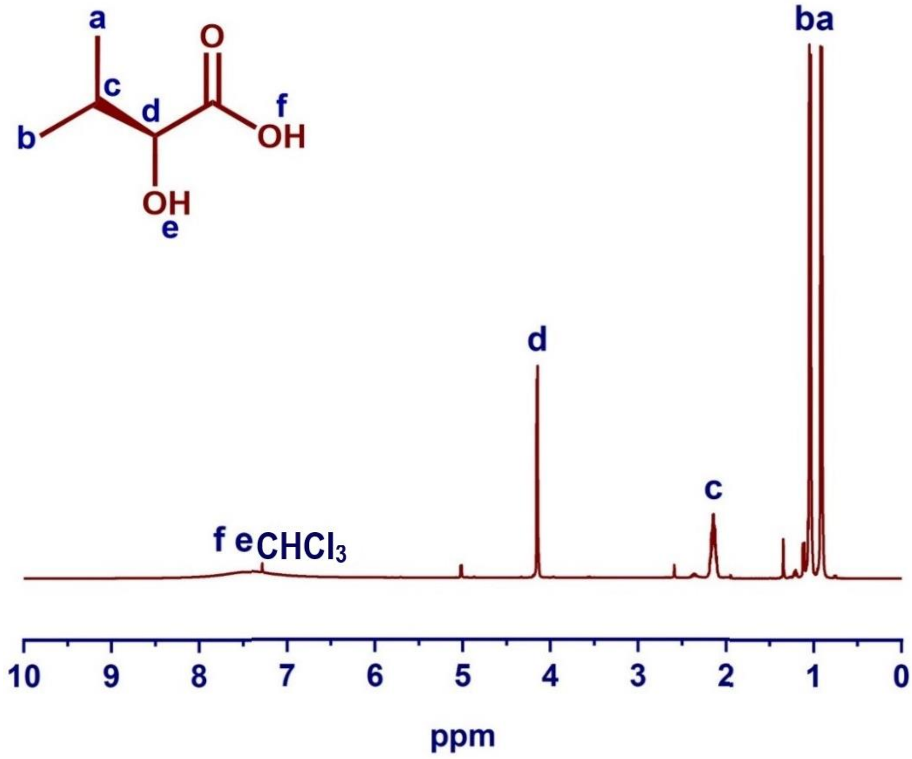

Figure S15. ^{1}H-NMR spectrum of L-2-hydroxy-3-methylbutanoic acid (IPHA) **4**

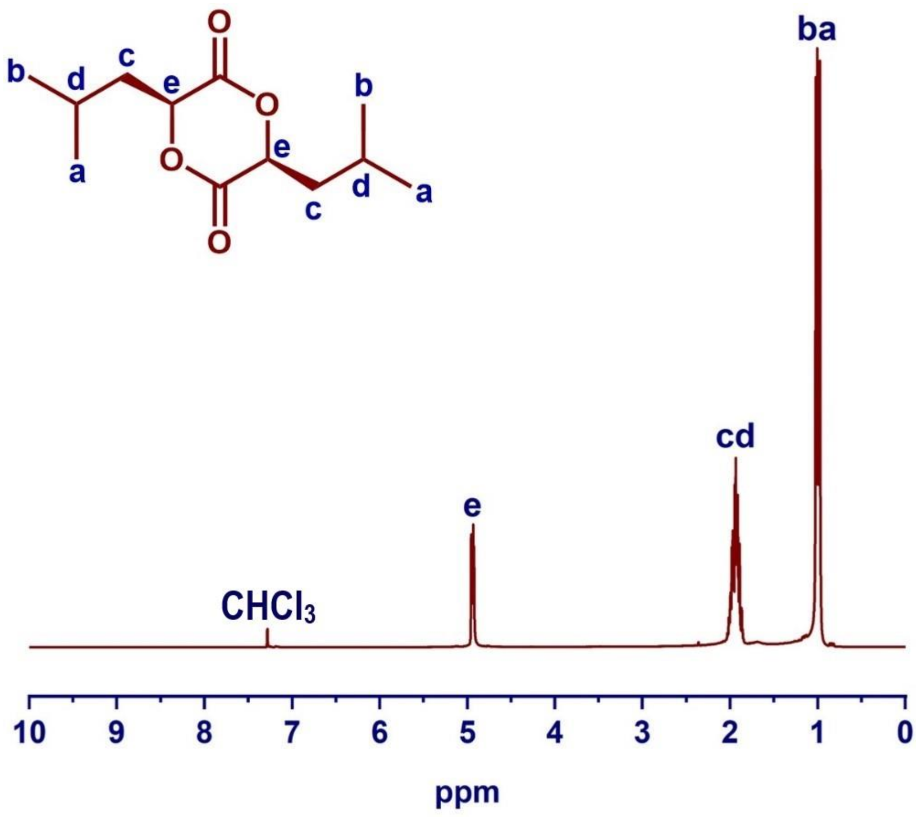

Figure S16. ^{1}H-NMR spectrum of L-3,6-diisobutyl-1,4-dioxane-2,5-dione (L-DIBG) **5**

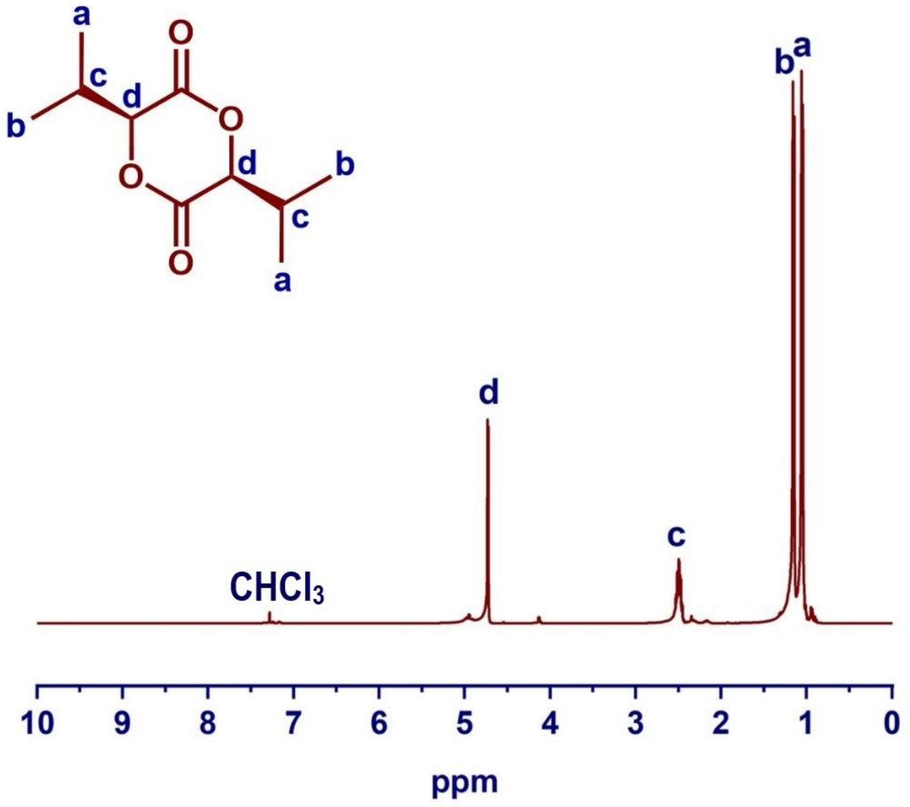

Figure S17. ^{1}H-NMR spectrum of L-3,6-diisopropyl-1,4-dioxane-2,5-dione (L-DIPG) **6**

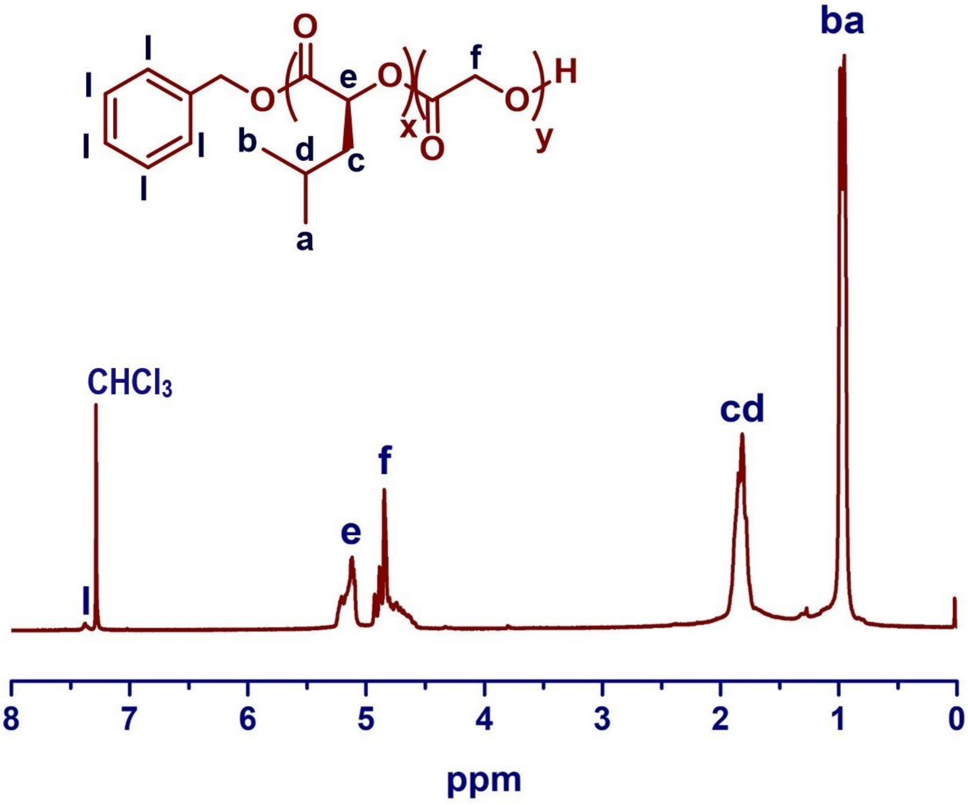

Figure S18. ^{1}H-NMR spectrum of PDIBG-PGA **9** copolymer

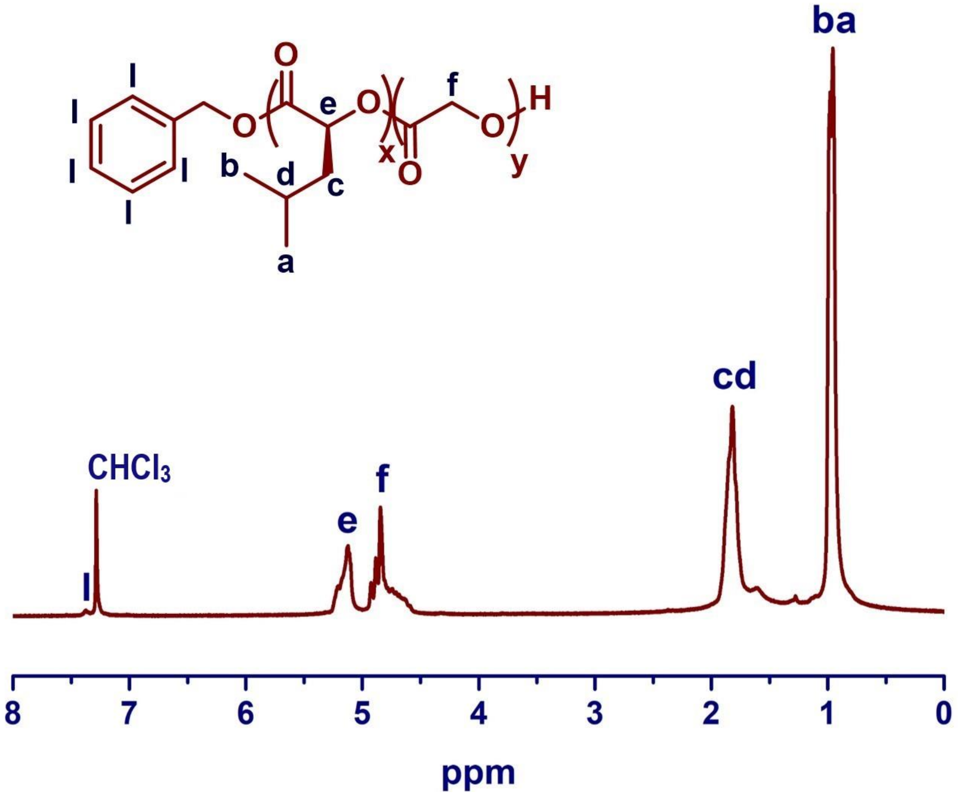

Figure S19. ^{1}H-NMR spectrum of PDIBG-PGA **10** copolymer

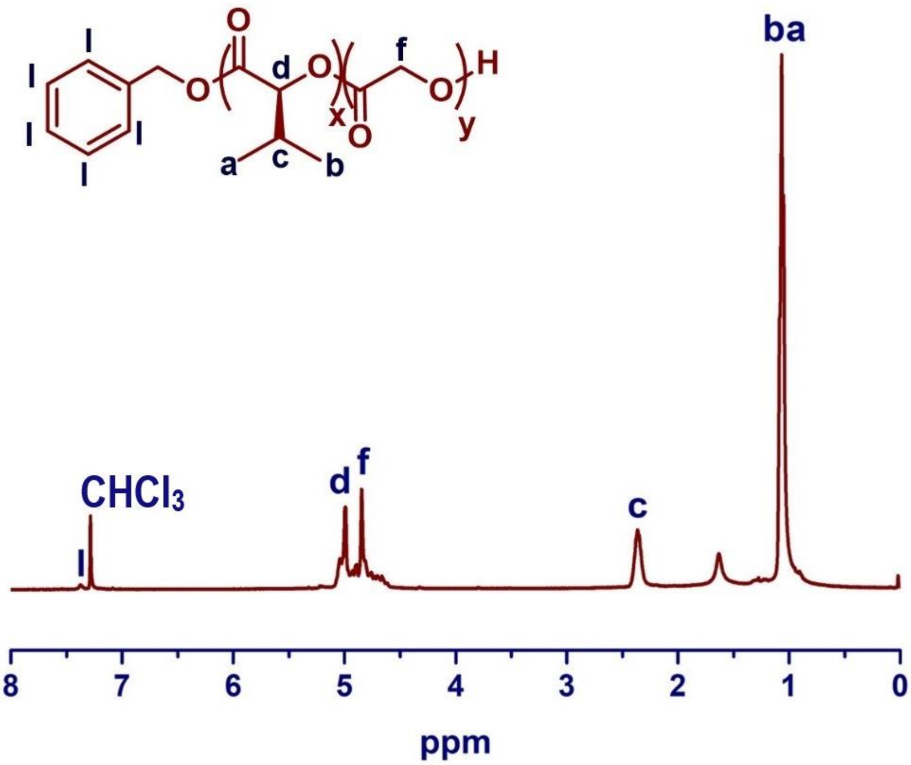

Figure S20. ^{1}H-NMR spectrum of PDIPG-PGA **12** copolymer

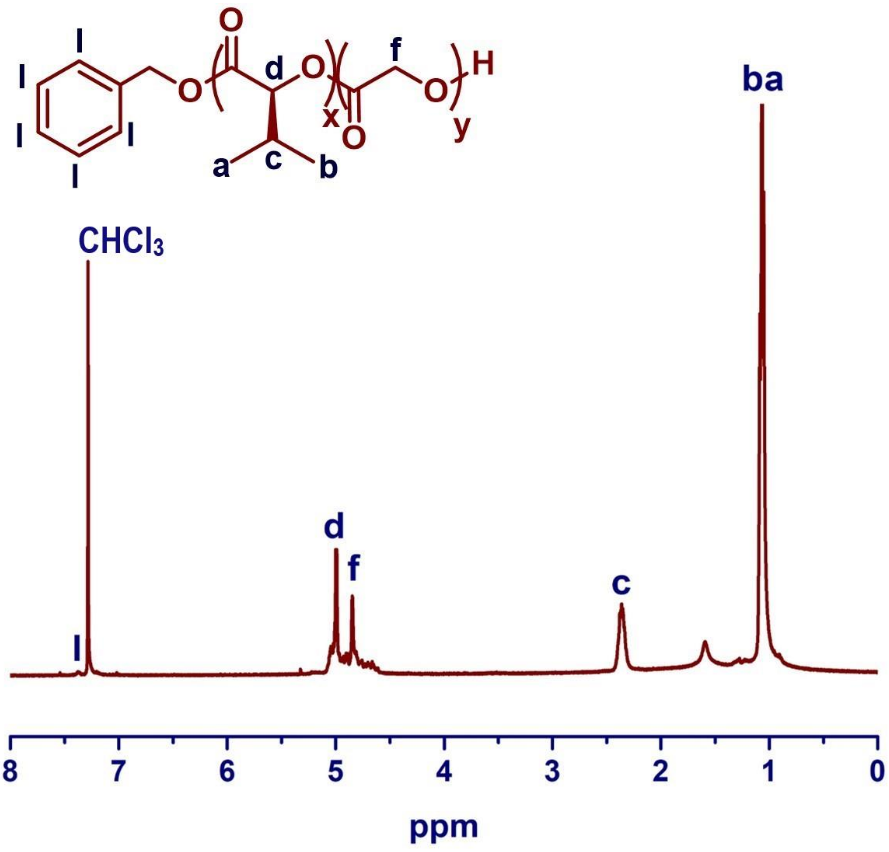

Figure S21. ^{1}H-NMR spectrum of PDIPG-PGA **13** copolymer

^{13}C-NMR Spectra

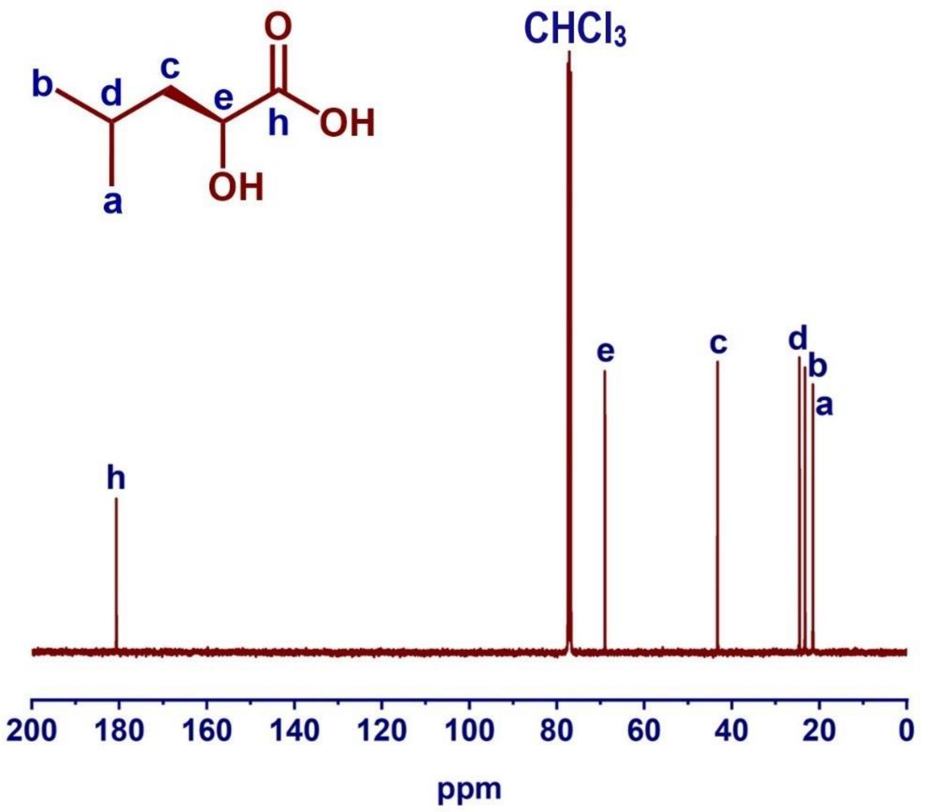

Figure S22. ^{13}C-NMR spectrum of L-2-hydroxy-4-methylpentanoic acid (IBHA) **3**

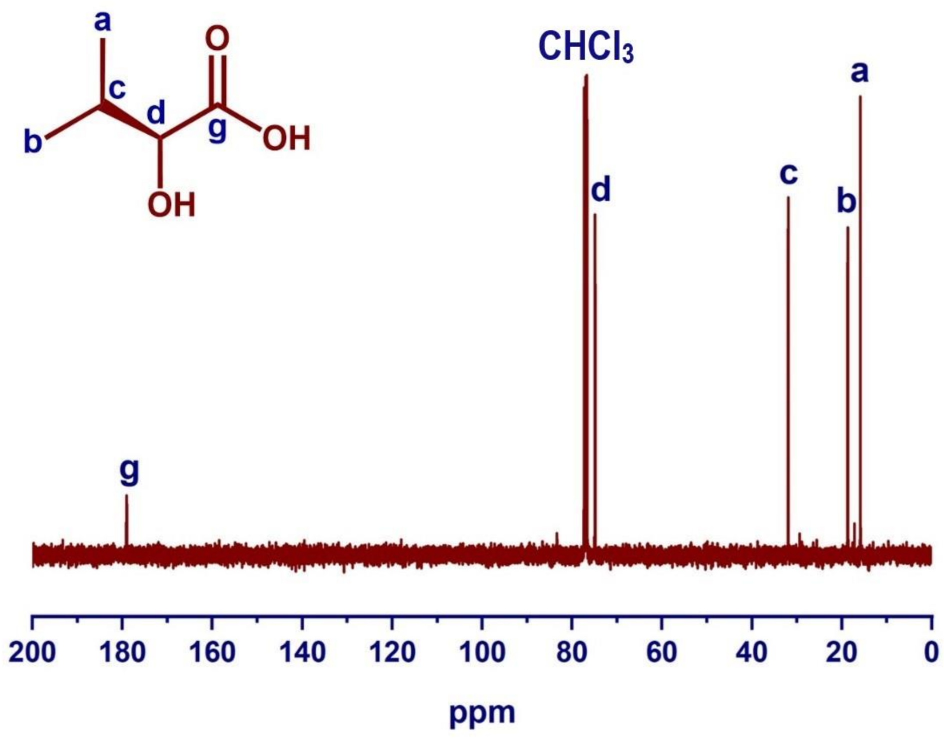

Figure S23. ^{13}C-NMR spectrum of L-2-hydroxy-3-methylbutanoic acid (IPHA) **4**

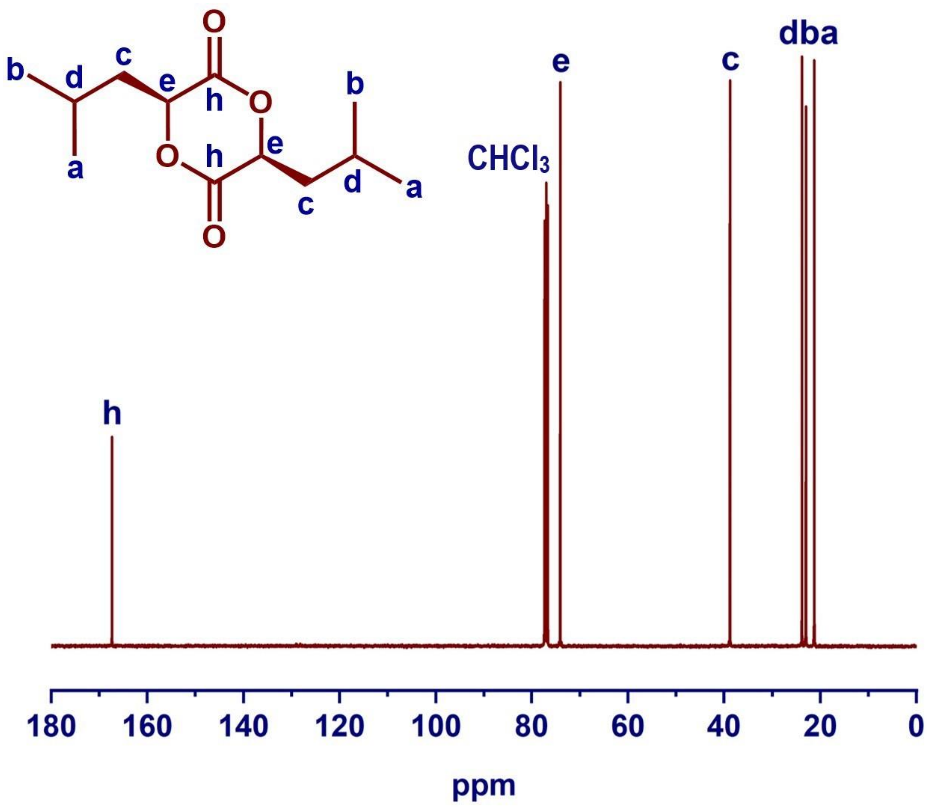

Figure S24. ^{13}C-NMR spectrum of L-3,6-diisobutyl-1,4-dioxane-2,5-dione (L-DIBG) **5**

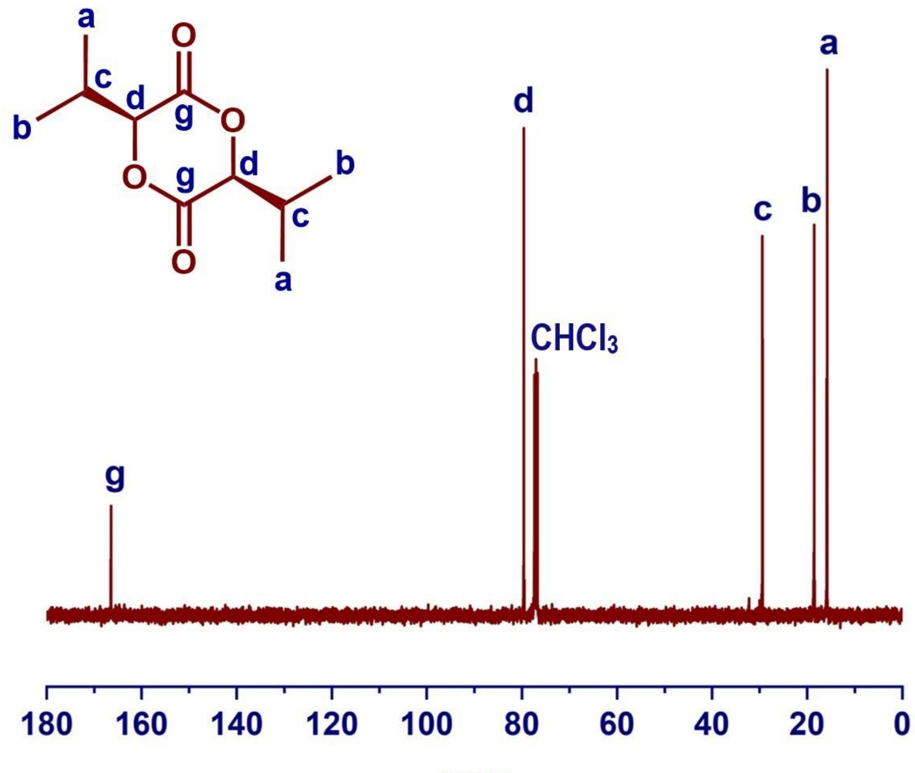

Figure S25. ^{13}C-NMR spectrum of L-3,6-diisopropyl-1,4-dioxane-2,5-dione (L-DIPG) **6**

REFERENCES

(1) Çetin, D.; Arıcan, M. O.; Kenar, H.; Mert, S.; Mert, O. Poly(asymmetrical glycolide)s: The Mechanisms and Thermosensitive Properties. *Macromolecules* 2021, *54* (1), 272-290.
(2) Arıcan, M. O.; Mert, O. Symmetrical substituted glycolides: methodology and polymerization. *Polymer Chemistry* 2020, *11* (27), 4477-4491.
(3) Arıcan, M. O.; Mert, O. Synthesis and properties of novel diisopropyl-functionalized polyglycolide–PEG copolymers. *RSC Advances* 2015, *5* (87), 71519-71528.
(4) Weidner, S. M.; Kricheldorf, H. R.; Scheliga, F. Ring-Expansion Copolymerization of l-Lactide and Glycolide. *Macromolecular Chemistry and Physics* 2021, *222* (3), 2000307.
